# A Bibliometric Survey of Paraffin/Olefin Separation Using Membranes

**DOI:** 10.3390/membranes9120157

**Published:** 2019-11-26

**Authors:** Débora Micheline Vaz de Miranda, Luciana da Silva Dutra, Débora Way, Nicolis Amaral, Frederico Wegenast, Maria Clara Scaldaferri, Normando Jesus, José Carlos Pinto

**Affiliations:** 1 Programa de Engenharia Química/COPPE, Universidade Federal do Rio de Janeiro, Cidade Universitária, CP 68502, Rio de Janeiro 21941-972, Brazil; dmiranda@peq.coppe.ufrj.br (D.M.V.d.M.); dway@peq.coppe.ufrj.br (D.W.); nicolis.amaral@yahoo.com.br (N.A.); fwegenast@peq.coppe.ufrj.br (F.W.); 2 Escola de Química, Universidade Federal do Rio de Janeiro, Cidade Universitária, CP 68525, Rio de Janeiro 21941-598, Brazil; ldutra@eq.ufrj.br; 3 Braskem S.A., Rua Marumbi, 1400, Campos Elíseos, Duque de Caxias 25221-000, Brazil; clara.scaldaferri@braskem.com (M.C.S.); normando.jesus@braskem.com (N.J.)

**Keywords:** membrane, olefin/paraffin, membrane technology, gas separation, bibliometry

## Abstract

Bibliometric studies allow to collect, organize and process information that can be used to guide the development of research and innovation and to provide basis for decision-making. Paraffin/olefin separations constitute an important industrial issue because cryogenic separation methods are frequently needed in industrial sites and are very expensive. As a consequence, the use of membrane separation processes has been extensively encouraged and has become an attractive alternative for commercial separation processes, as this may lead to reduction of production costs, equipment size, energy consumption and waste generation. For these reasons, a bibliometric survey of paraffin/olefin membrane separation processes is carried out in the present study in order to evaluate the maturity of the technology for this specific application. Although different studies have proposed the use of distinct alternatives for olefin/paraffin separations, the present work makes clear that consensus has yet to be reached among researchers and technicians regarding the specific membranes and operation conditions that will make these processes scalable for large-scale commercial applications.

## 1. Introduction

Cost-effective gas separation technologies are required in many important industrial applications to withstand the harsh operating conditions of a petroleum refinery [1]. Particularly, cryogenic distillation is the commonest technology employed for purification of gaseous streams, despite the high costs of cryogenic operations and equipment. For this reason, the use of separation technologies based on adsorption, absorption and membranes has been encouraged lately in order to reduce energy costs and improve gas separation efficiencies. However, some inherent characteristics of these technologies (such as sensitivity to impurities, degradation of separation materials, narrow ranges of operation conditions, among others) still negatively affect their full acceptance and prevent the replacement of the expensive cryogenic distillation techniques [1,2]. For example, absorption columns may be unable to handle very high and low flow rates, demand high capital investments and require high operational costs. Besides, solvent regeneration may lead to significant energy consumption, while unavoidable solvent loss increases the environmental impacts of the process, due to solvent emissions, among other causes. On the other hand, adsorption processes may be subject to kinetic limitations and loss of sorption capacity over multiple adsorption cycles. Additionally, adsorbent particles may present thermal, chemical and mechanical stability issues, which can lead to particle erosion and degradation over the operation cycles. In turn, membranes usually suffer from poisoning by impurities present in the feed streams and most membrane processes have not been validated industrially yet [3]. More specifically, facilitated transport membranes usually lack stability due to the loss of the carrier during the operation or inactivation of the carrier in the presence of impurities [4]. As carbon molecular sieves are brittle, these alternative materials require careful handling, and may be much more expensive than polymeric membranes [5]. Similarly, alternative ceramic and zeolite membranes can also be subject to poisoning by minor components present in the feed stream [6]. Finally, most polymeric membranes do not resist severe operating conditions (such as high temperatures) and the presence of many organic vapors and solvents [7], showing low selectivity to olefin/paraffin separation [8].

Despite the previous remarks, the main advantage of using membranes for gas separation is the fact that membrane processes can allow for process intensification, leading to significant reduction of production costs, equipment size, energy consumption and waste generation. Besides, membranes can usually be provided by manufacturers and installed in plant sites in modules, allowing for easier fitting to the particular process demands [5]. Consequently, membrane separation processes have gained industrial acceptance and compete favorably with other consolidated operations in some specific niches of gas separation. In particular, the use of membranes for separation of N_2_/O_2_ [5], CH_4_/CO_2_ [9,10,11,12,13,14,15,16,17,18,19,20,21,22,23,24,25,26,27,28,29,30,31,32,33,34,35,36,37,38,39,40,41,42,43,44,45,46], N_2_/CO_2_ [47,48,49,50,51,52] and H_2_/CO_2_ [11] streams has become industrially attractive and commercially available. Air Products, Generon, Honeywell UOP and Schlumberger are some of the players that are involved with the production of membranes for gas separation. Therefore, there are many indicatives that membrane technology has vast potential to overcome energy issues encountered in cryogenic distillation processes and that membrane technologies will deserve deeper technical and scientific attention in the near future [53]. 

Otherwise, one of the main difficulties of most membrane technologies is the simultaneous obtainment of high separation selectivities and high permeabilities (or process productivities) [54], which many times hampers the commercial use of membrane technologies. This undesired effect is related to the fact that there is an upper bound on the trade-off between membrane permeability, which limits flow rates, and the selectivity, which limits the quality of the separation process [54]. According to Robeson [55,56], the inverse relationship between selectivity and permeability can be observed for most pairs of permeable gases and polymeric membranes, which leads to a practical limit named as the Robeson’s Upper Bound [56], which can be correlated with the molecular characteristics of the permeating gas and of the polymer used to manufacture the membrane barrier. These correlations can be eventually used for design of membrane materials and improvement of gas separation processes [57].

For this reason, development of effective membrane separations can be rather complex and normally requires a great deal of research and development in order to deliver acceptable commercial performances [58]. As a consequence, it is not surprising to observe that different types of membrane technologies have been proposed throughout the years and that the field continues to evolve [8,59].

Olefins are among the most important products of a petrochemical industry because they are used as intermediates for manufacture of many other chemicals [60]. For this reason, olefin/paraffin separation is one of the most important processes in oil refineries. As a matter of fact, even very small improvements in this area may exert an enormous financial impact on the economical performances of refineries. Additionally, the growing demand for olefins, such as ethylene and propylene, especially in emerging consumer markets, creates new opportunities for technologies that can lead to increase of olefin supply and reduction of production costs [8,59,61]. As a consequence, membrane separations can constitute excellent alternatives to expensive distillation processes [1,2,5].

Despite that, membrane-based processes still do not find full industrial acceptance in the field of paraffin/olefin separations, which encourages the conduction of bibliometric analyses to characterize the maturity and the main bottlenecks of this technology. Particularly, Table 1 and Table 2 summarize the membranes and the respective separation mechanisms that have been presented most often as possible solutions for separation of olefin/paraffin streams, as well as the key challenges that affect the progress in this area.

Based on the previous paragraphs, the main objective of the present work is the development of an extensive bibliometric survey regarding the use of membranes for gas separations in petrochemical processes. In particular, it is intended to characterize the degree of maturity and the main bottlenecks of processes used for separation of light hydrocarbon mixtures containing homologous series of paraffins (methane, ethane, propane, among others) and olefins (ethene, propene, among others), focusing on ethane/ethylene and propane/propylene mixtures. Therefore, the present study also reports the membranes that are used most often, the usual process configurations, the operating conditions and the stability of the applied materials, as described in the available scientific and technical literature.

## 2. Data Sources and Methodology 

The investigation of paraffin/olefin separations using membranes was performed with help of electronic search tools including Google Scholar, Google Patents, USPTO and EspaceNet. At first, screening searches were performed using the expressions “*gas separation membrane(s)*” and “*olefin/paraffin separation membrane(s)*”, placed anywhere in the text. Afterwards the searches were refined with help of more specific expressions located in the title or abstract, including “*membrane(s) separation(s)”* AND “*olefin(s)*”; “*membrane(s) separation(s)”* AND “*paraffin(s)*”; “*membrane(s) separation(s)”* AND (“*ethane*” OR “*C2H6*”); “*membrane(s) separation(s)”* AND (“*ethene*” OR “*ethylene*” OR “*C2H4*”); “*membrane(s) separation(s)”* AND (“*propane*” OR “*C3H8*”); “*membrane(s) separation(s)”* AND (“*propene*” OR “*propylene*” OR “*C3H6*”). Searches were performed considering the papers published until August 2019. Then, the obtained documents were downloaded, analyzed and eventually accepted for this bibliometric survey, as described in the following paragraphs. After reading and analyzing the selected documents, additional relevant references not captured by the electronic searches were also included in the set of accepted documents. For the purposes of the present investigation, accepted documents were also used to provide information regarding the fifteen information categories listed in Table 3, which were analyzed as presented in Section 3.

In Table 3, categories #1 and #2, institutions and countries, were reported considering the affiliation of the corresponding author. Category #5, number of citations of the analyzed document, is important because it can be used to evaluate the relative relevance of the publication. Categories #6 to #10 (gas feed compositions, selectivities or separation factors, permeabilities, operation temperatures and operation pressures) provide information about the reported operation conditions. Categories #11 regards the processed gaseous streams, while category #12 describes the material used to manufacture the membranes, classified as: 12.1) polymer: polymer membranes that do not include the use of carriers or other components; 12.2) zeolite: zeolite membranes that do not include the use of carriers or other components; 12.3) facilitated transport (polymer): polymer membranes that include the use of carriers to facilitate olefin permeance through the membrane; 12.4) facilitated transport (liquid): liquid membranes that are supported by different kinds of materials, usually containing a metal carrier to facilitate the olefin transportation through the liquid solution (typically AgNO_3_ or AgBF_4_); 12.5) facilitated transport (hybrid): membranes that combine two or more types of materials, as composites or mixtures of polymers and inorganic matrices, and use metal carriers to facilitate olefin transportation; 12.6) CMS (carbon molecular sieve): membranes composed of pyrolyzed polymers; 12.7) MOF (metal-organic frameworks): organic or inorganic membrane matrices where metal compounds are anchored to facilitate transportation; 12.8) others: including absorbents, adsorbents, hybrid systems (membranes that combine two or more types of materials as composites or mixtures of polymers and inorganic matrices) and ionic liquid membranes. Category #13 reports the metals used to facilitate the olefin transportation, whenever applicable. Category #14 describes the geometric features of the membrane separation equipment, classified as flat sheets, spiral wounds and hollow fiber membranes. In this category, adsorption and absorption columns were also considered, as these separation strategies also constitute interesting alternatives for paraffin/olefin separations. Finally, category #15 reports the lifetime of the analyzed membranes, one of the main concerns in the field [66].

## 3. Results

The preliminary screening search regarding “membrane gas separation” resulted in 5660 documents. After initial filtering, as mentioned in the previous section, the final set of documents comprised 300 papers (Table S3) published since the 1960s, which are analyzed below in accordance with the categories described in Table 3, which were analyzed as presented in Section 3.

From this set of documents, 236 papers regard membrane separations of gaseous streams contain studies related to mixtures between paraffins and olefins, while 64 papers regard membrane studies that analyze paraffin and/or olefin permeation (not necessarily considering their mutual separation). Thus, considering the proposed search methodology, it seems correct to say that membrane olefin/paraffin separations represent approximately 5% of the total number of papers published in this field, indicating that membrane olefin/paraffin separations do not constitute the mainstream of the area and suggesting that this technology is still under development, as reinforced in the next sections.

### 3.1. The Annual Distribution

**Figure 1:**
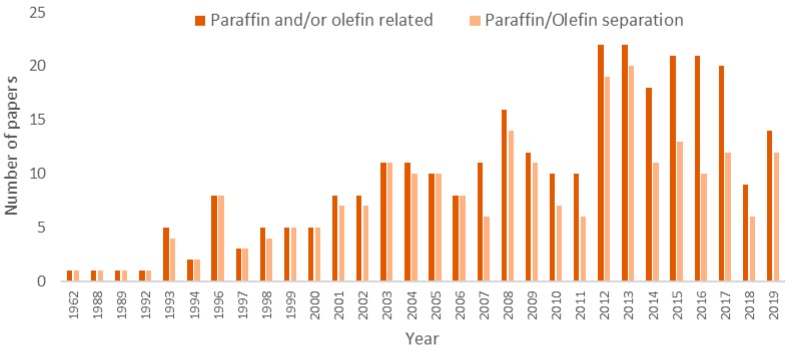
Annual production of papers in the field of membrane paraffin/olefin gas separations.

**Figure 2:**
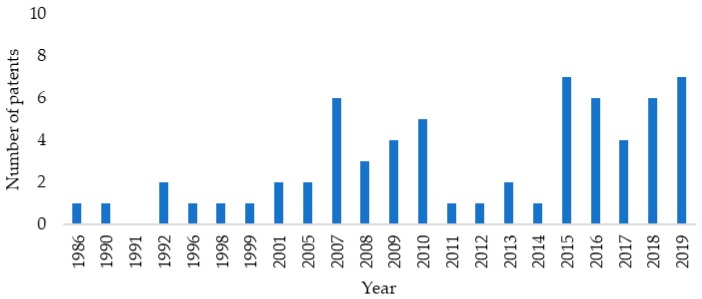
Annual production of patents in the field of membrane paraffin/olefin gas separations.

Figure 1 and Figure 2 present the annual distribution of publications and patents in the field of olefin/paraffin membrane separations. It must be highlighted that the first document in the analyzed field was published in 1962, regarding the separation between pentene and pentane by adsorption in solid matrixes, using charcoal as adsorber by Kellogg Company [72]. Then, in 1988, ExxonMobil published a study regarding the ethylene/ethane separation through complexation with cuprous diketonate in alpha-methyl styrene [73]. According to the methodology used in the present study, the number of papers and patents published in the field is relatively small and has not grown significantly through the years, being subject to periodic oscillations that are related to economical constraints, such as the development of shale gas technologies and the oscillation of prices of oil and gas [74]. In more recent years, the rate of scientific production increased to about 20 papers per year, due to the arousal of new technologies, such as metal-organic-frameworks (MOF) [75,76,77,78,79,80] and carbon molecular sieves (CMS) [81,82,83,84,85,86,87], which became more visible after 1995. When one considers the significant economic advantages that can be attained with membrane separation processes and the relatively small number of publications in this field, one can probably conclude that bottlenecks still inhibit the full industrial development of the technology.

Thomas Graham was the first to propose a description for the sorption-diffusion process in 1866 [88]. Between the years of 1940 and 1950, Barrer, van Amerongen, Meares, and others, built the fundamentals of the modern theory of gas permeation, incorporating advances of polymer sciences. The sorption-diffusion model for gas permeation, developed by Graham, continues to be a widely accepted model for the transport of gases through membranes. However, membrane manufacturing technologies have not been sufficiently robust to make membrane systems useful for separation of paraffins and olefins from gaseous streams in commercial scale, which partially justifies the lack of scientific production in the area from 1963 to 1988 [89]. Despite that, in 1989 an important paper about paraffin/olefin separations using membranes entitled “*Separation of ethylene from ethane by a flowing liquid membrane using silver nitrate as a carrier*” was published, reporting a new type of liquid membrane with the ability to overcome the instability and the low permeability of thin-layer liquid membranes [90].

### 3.2. The Scientific Journals Distribution

The distribution of publications in scientific journals is shown in Table 4, for journals that published 5 or more papers in the analyzed area. As one can see, publications have been concentrated in relatively few journals, with significant concentration in Journal of Membrane Science (101 papers or 34% of the analyzed set) and Industrial and Engineering Chemistry Research (32 papers or 11% of the analyzed set). The high quality of the journals (with IF values above 1.1) must be highlighted and indicates that this issue is regarded as relevant by the academic community. On the other hand, the extremely high concentration of papers in few journals indicates that relatively few aspects of the analyzed problem have been addressed by the scientific community, with emphasis on the production and characterization of membranes used to perform the separation of the gaseous streams.

### 3.3. The Country and Institutions Distribution

**Figure 3:**
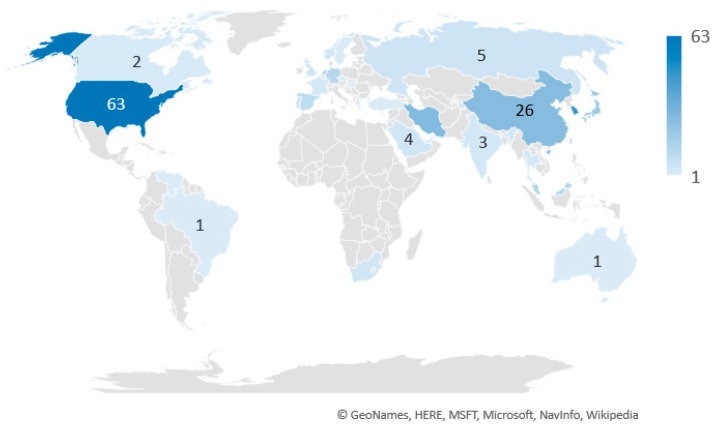
Country distribution of papers in the field of membrane paraffin/olefin gas separations.

Figure 3 and Table 5 show the country distribution of papers in the analyzed field. As a whole, the papers involved 130 institutions of 32 distinct countries, indicating the widespread interest in this area, although 6 countries concentrate more than 60% of the total number of documents of the area. Among these 6 countries, the concentration of papers in USA, Iran and China is probably related to the fact that these countries are major oil producers and present well-established refining complexes, while the concentration of papers in Netherlands and Japan probably indicates a more genuine interest of the involved institutions in the technical aspects of membrane separation technologies.

The participations of USA and Korea are also prominent in the patent area, concentrating almost 80% of all patents published in this field. When compared to published papers, France, Canada, Portugal and Saudi Arabia can be regarded as relevant developers of patents in the analyzed area (5% of the total number of published papers and 24% of the total number of patents), despite the lower number of patents, as shown in Table 6.

The considerable contribution of South Korea, responsible for 16% of the total number of publications, may represent the efforts made by the South Korean government to support investments in research and development (RandD) related to more sustainable processes [91]. It is interesting to note that, although Japan and China are among the most productive countries in the field, Japanese and Chinese institutions are not among the most productive ones, as observed in Table 7, which indicates that Japanese and Chinese productions are shared with other international institutions.

Table 8 presents the ranking of patent applicants. ExxonMobil, UOP, Institut Français du Petrole, Industry-University Cooperation Foundation Hanyang University and Korea Institute of Science and Technology apparently stand out as top patent applicants for paraffin/olefin separations using membranes. Despite that, the patent production does not reflect the availability of large-scale commercial facilities, although it is true that pilot plants are currently under operation in different institutions, as recently reported by Dow Chemical. It is worth mentioning that the authors of patents filed by the Korea Institute of Science and Technology are the same authors that published many of the Korean papers, which may indicate that this innovative activity is not necessarily connected with actual commercial manufacture of new membrane products [92,93,94,95,96,97,98,99,100].

### 3.4. The Most Cited Papers

Table 9 shows the most cited papers in the investigated field. As one can observe, the most cited papers describe the use of different membrane materials for separation of gaseous streams that contain paraffins and olefins. This probably shows that the scientific research in this area is still driven by the necessity to develop new materials that can improve the efficiency of membrane paraffin/olefin separations. Still, it is important to note that among the most cited documents, three deal with a relatively recent membrane type, which may be an indicative that MOFs are being seen by the scientific community as promising materials for gas stream separation, specifically considering the paraffin/olefin mixture.

### 3.5. The Separated Streams

**Figure 4:**
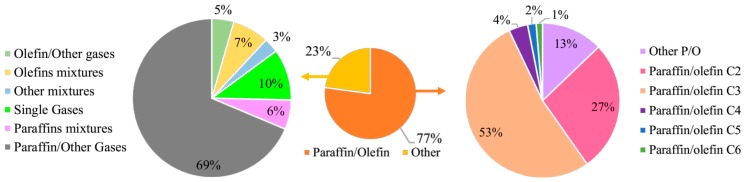
Streams reported in papers regarding paraffin/olefin membrane separations.

Based on the adopted search criteria, 322 distinct streams have been reported in the literature, being that 248 streams of them contained mixtures of paraffin/olefin and 74 of them contained other gaseous components. In the last case, membrane permeabilities of pure gaseous streams, such as single paraffins or single olefins; separations of streams containing paraffin mixtures (butane/methane, butane isomers, pentane/octane, and propane/methane, for example) or olefin mixtures (butenes, di-olefin/mono-olefin, 1-hexene/1,5-hexadiene, acetylene/ethylene, for example); and separations of olefins or paraffins from other gases, such as N_2_, air, argon, H_2_S, CO, H_2_, and CO_2_, have also been reported, as summarized in Figure 4.

It is important to notice that the number of studies involving separations of mixtures of paraffins with other gases is expressive. Almost 85% of the publications reported in the field of membrane gas separations regard mixtures of paraffins and CO_2_, especially methane and CO_2_, due to the importance of this gaseous stream for the oil production industry and necessity to remove carbon dioxide from natural gas during oil production and enhanced oil recovery [106]. 

Although the present work has emphasized the separation of ethane/ethylene and propane/propylene streams, studies with other olefin and paraffin streams, strongly associated with gaseous effluents from petrochemical industries, have also been evidenced. This may be attributed to the necessity to enrich and utilize certain valuable chemicals, such as isobutene (in isobutene/isobutane mixtures), 1,3-butadiene (in 1,3-butadiene//n-butane mixtures), 1-heptene (in heptene/heptane mixtures), 1-hexene (in hexene/hexane mixtures), 1-pentene (in pentene/pentane mixtures), cyclohexene (in cyclohexene/cyclohexane mixtures), among others, with help of technologies that can be more efficient than conventional distillation processes. 

The collected data set was filtered and is available as Supplementary Material. Table S1 shows the reported membranes and the separation factors for some gaseous streams containing paraffins and/or olefins. Table S2 displays some papers that present detailed geometric configurations and operation conditions for olefin/paraffin membrane separations, with emphasis on separations of ethane/ethylene and propane/propylene streams.

### 3.6. The Used Membranes

Membranes have been successfully employed for separations of many liquid streams [107] and many specific gaseous streams, such as mixtures of H_2_, CO_2_ and CH_4_, known as “fast gases” or gases with high permeations [108]. AirLiquide^©^, Schlumberger^©^, Generon^©^, AirProducts^©^, among others, are companies that provide commercial membranes for separation of these fast gases. However, membrane paraffin/olefin separation technologies are not consolidated yet, so that process development is still in the pilot scale phase in most cases. Particularly, researchers observed a long time ago that addition of a carrier to the membrane material might lead to higher selectivities and permeabilities, constituting a major advance in the area [4,109]. As observed in the analyzed papers, 55% of the papers used some type of carrier to facilitate the separation process, indicating a tendency to adopt Facilitated Transport Membranes (FTM) for paraffin/olefin separations [8]. The carrier is expected to interact with one component of the gaseous stream (usually the olefin), increasing the apparent solubility and permeability of the compound in the membrane material [8,110]. Usually, the carrier contains a metal atom with free valences that make possible the electronic interaction with the electronic cloud of the carbon double bond of olefins [8,111,112,113]. 

**Figure 5:**
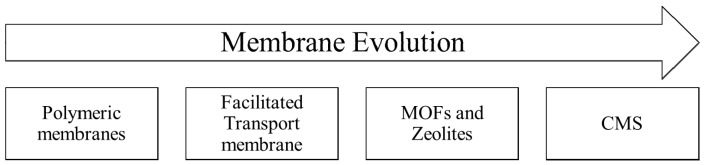
Schematic representation of the evolution of membrane systems used for paraffin/olefin separations.

**Figure 6:**
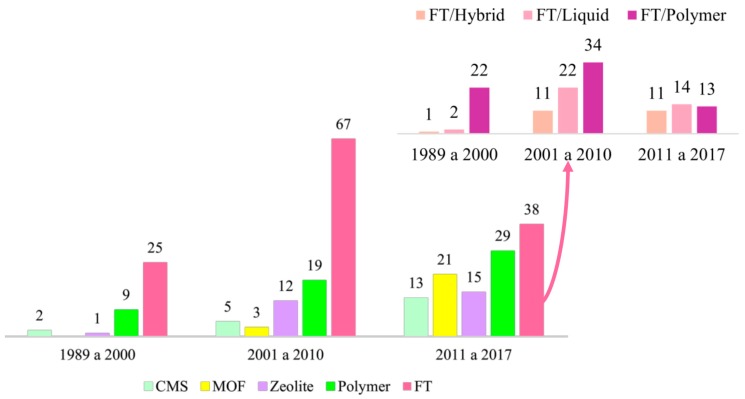
Types of membranes reported in papers regarding paraffin/olefin membrane separations.

Figure 5 presents the schematic representation of the evolution of membrane technology. Facilitated transport membranes (where the use of a carrier increases the membrane selectivities) initially displaced conventional polymer membranes because of the best separation performances. However, the search for even better separation coefficients and dynamic stability opened room for introduction of zeolites, carbon molecular sieve membranes (CMSs) and metal-organic frameworks (MOFs). Although the use of CMSs, MOFs and zeolites for olefin/paraffin separations was modest until the 2000s, as observed in Figure 6, these latest technologies have been extensively studied for 15–20 years and seem promising for paraffin/olefin separation applications. Despite that, it is still necessary to enhance important properties, such as mechanical resistance, performance stability and production cost, for large-scale industrial applications to become technically and economically viable in the field of membrane paraffin/olefin separations.

**Figure 7:**
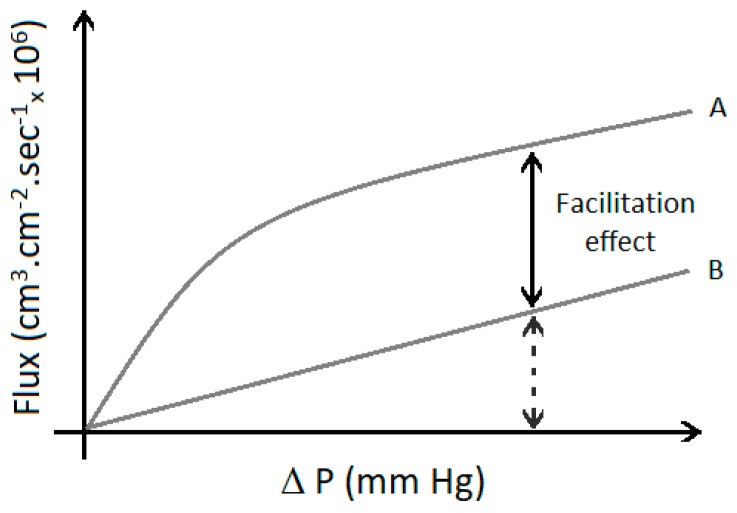
Effect of facilitated transport through the membrane: solute diffusion using (A) FTM and (B) carrier-free membrane. (Adapted from [119].).

Facilitated transport has been the most cited mechanism in the analyzed literature, even after the advent of new technologies. Figure 6 indicates that most of these studies (44%) proposed the addition of a carrier agent into a polymer matrix, leading to synergetic effects between the solution-diffusion process and the chemical interaction between the olefin and the membrane through complexation of the carrier agent (as illustrated in Figure 7). Table S2 presents the relevant data collected and the main FT membranes used for olefin/paraffin separations.

FTM was originally introduced by Scholander in 1960, for purification of O_2_ streams [114], and has been intensively studied since then [114,115]. FTM enables the selective transportation of molecules and explores reversible chemical interactions between the target species and the active sites (carriers) to accomplish the transport of the target molecule through the membrane matrix, leading to enhanced membrane permeability and selectivity. Meanwhile, other species that do not react with the active sites permeate through the membrane only through the usual solution-diffusion mechanism [116,117,118]. Figure 6 illustrates the effect of the carrier on the transport through the membrane.

GALIZIA and co-workers (2017) [116] stated that it is possible to achieve superior separation properties using membranes based on facilitated transport mechanisms for many blends that are difficult to separate, such as mixtures of paraffins and olefins and of aromatic compounds [120]. Most FTM separation processes usually make use of silver as the carrier, which can interact specifically with the olefin. The ability of olefins to form reversible organometallic complexes with some transition metal cations, such as Ag^+^ ions, ensures the separation process [1,121]. Based on the olefin complexation theory, FAIZ and LI (2012) [65] observed that the use of metals for complexation with olefins could lead to efficient separation of gaseous mixtures of paraffins and olefins. The high stability of metal-olefin complexes can be explained by interactions between the atomic orbitals of the metallic atom and the molecular orbitals of the olefin molecules, as postulated by Dewar using the Molecular Orbital Theory [105]. The bonds formed in the complex are stronger than Van der Waals forces, but still sufficiently weak to break by temperature increase or pressure reduction [121,122], making the reversible reaction possible.

In order to increase the reversible reactivity of the transition metal ion with olefins, the anion of the transition metal plays an important role in determining the intensity and the rate of the interaction between the carrier and olefins. Due to the lower lattice energy of the transition metal salt, the anion forms a weak ionic bond or ion pair with the cation and the salt can be easily dissolved in solutions. Therefore, it is preferable to select a transition metal anion that possesses low lattice energy in respect to the metal cation [123]. For facilitated transport to occur, the lattice energy of the transition metal salt must be preferably smaller than 1000 kJ/mol, reducing the tendency of the anion of the transition metal salt to form a strong ion pair with the cation [123,124].

Based on criteria reported usually in the literature for effective FTM processes, including electronegativity, lattice energy and intensity of π-complexation between metals and olefins, silver salt has been largely selected as the most appropriate carrier for facilitated transport of olefins. The commonest generalized and overall reaction scheme for the transport of olefins across the membrane is shown in Equation (1) [1,117,118,123]:

*Olefin* + *Ag*^+^ ⇌ [*Olefin.Ag*^+^] (1)

RAVANCHI (2015) [118] studied the influence of carrier concentration on propylene/propane separation using hydrophilic poly(vinylidene difluoride) (Ag^+^/PVDF) flat sheet membranes. The author concluded that it is important to consider three parameters simultaneously for process design: trans-membrane pressure, carrier concentration and effect of feed composition on the separation factor. It is important to highlight that facilitated transport is a combination of two processes: absorption (on the feed side) and stripping (on the permeate side). Increasing the pressure favors absorption and decreasing the pressure favors stripping. Thus, increasing the feed pressure increases the absorbed olefin on the feed side. Due to the pressure difference between the feed side and the permeate side, the olefin complex must be degraded on the permeate side. Therefore, increasing the transmembrane pressure enhances the driving force for separation. Besides, the separation factor and olefin permeability can be increased when higher concentrations of Ag^+^ ions (ranging from 5 to 20 wt%) are used. Table 10 presents the membranes and respective selectivities reported to separate olefin/paraffin employing facilitated transport mechanism.

**Figure 8:**
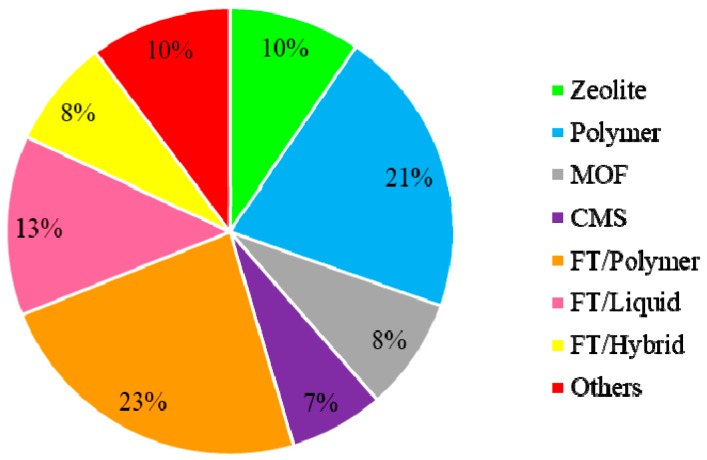
Distribution of membrane technologies used for separation of gaseous paraffin/olefin mixtures.

When compared to FT, the use of other technologies (Figure 8) is relatively less frequent, including the use of adsorbents [72,73,105,210,211,212], liquid membranes [41,48] and hybrid membranes [17,29,213,214], which were grouped as “Others” and concentrate 10% of the papers. The relatively large number of alternative membrane technologies indicate that researchers and technicians are still searching for an efficient and viable membrane system for separation of gaseous paraffin/olefin mixtures.

In 1996, it was reported that carbonized membranes produced with different materials might present higher permeabilities and selectivities than the non-carbonized precursor polymers [81]. Table 11 presents selectivities, permeabilities and operating conditions reported for gas separations using CMS membranes. When the numbers presented in Table 11. Reference values reported for gas separations with help of CMS membranes are compared to each other, it becomes possible to observe the high variability of the reported results, which makes difficult the definition of operation conditions and performance indexes for these systems.

The uses of zeolite and MOF membranes for separation of gaseous paraffin/olefin streams are presented in Table 12. The separation mechanism of the molecules is based mainly on the molecular sizes and shapes (geometrical selectivity) [52]. The studies have shown superior selectivity in MOF membranes. Special attention must be given to the membrane MIL-100(Fe), prepared in lab-scale with BET surface area of 2558 m^2^·g^−1^, which provided selectivities of 111 and 70 at 1 kPa and room temperature for ethylene/ethane and propylene/propane separations, respectively [225].

**Figure 9:**
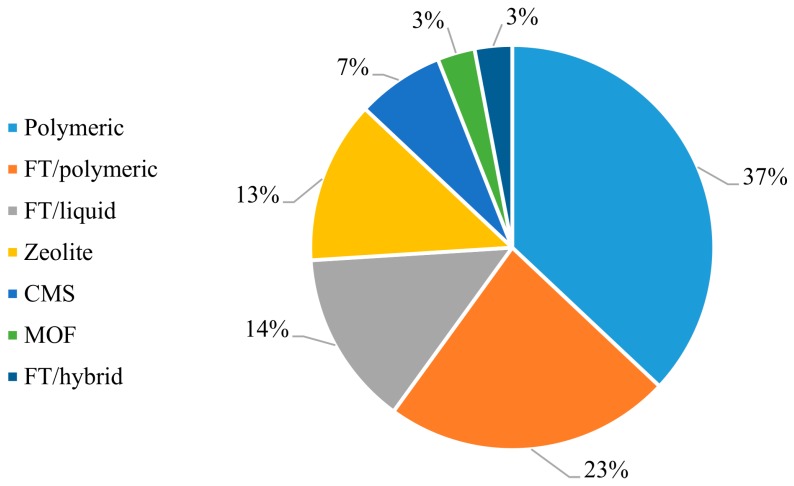
Types of membranes for paraffin/olefin separations.

As also observed in the published papers, the considerations regarding the membranes type in the deposited patents, as observed in Figure 9, show that polymers and polymers combined to metal carriers represent around 60% of the used materials. Also, other types of membranes were observed. CMSs, MOFs and zeolites depict almost 25% of the read patents. Facilitated transport and solution-diffusion are the most separation mechanisms, nearly 80%. Silver represents 82% of all the carriers used, however, gold and copper have been exploited [244]. As can be noticed, silver dominates the studies as also observed for the paper bibliometric analysis. 

However, zeolites [245], carbon molecular sieve [246] and metal-organic framework [247] membranes began to appear as interesting alternatives to overcome the separation limit traditionally observed for polymeric membranes.

The analyses of patents that regard olefin/paraffin separations apparently indicate that the development of new stable, highly permeable and highly selective facilitated transport membranes constitutes a critical issue for the future success of the technology, as commercial players are still searching for improvements that will make FTMs more attractive and viable [248,249,250,251,252,253]. It is also important to highlight that most feed streams reported in published patents contain simple mixtures of pure gases, so that the membrane performances have rarely been validated with real gas mixtures, meaning that deactivation of FTMs have probably been underestimated in most documents. Finally, actual industrial applications have not been properly documented in available patents, illustrating the scalability problems of the technology. Despite that, given the possible benefits obtained by overcoming the disadvantages usually associated with the conventional gas separation methods, it is certain that additional studies and investments will be performed to make the technology more viable and ready for commercial application.

### 3.7. The Carrier Agents

**Figure 10:**
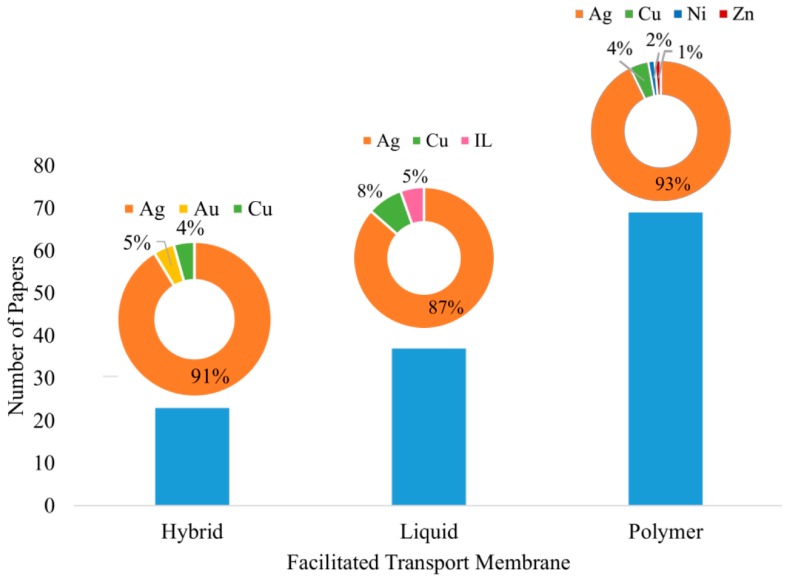
Distribution of carriers used for separation of gaseous paraffin/olefin mixtures in FTM processes.

Figure 10 presents the relative frequency of carriers reported in the literature. As one can observe, silver concentrates almost 90% of the papers published in the field of FTM. It must be noted that neutral Ag-nanoparticles have also been used as carriers in FTM processes. As reported in some documents, neutral Ag-nanoparticles are chemically stable, present excellent long-term performances and can lead to high selectivities and permeabilities [249]. Particularly, Campos et al (2018) [59] presented a critical analysis regarding the current state of development, the possible applications and the unstable nature of FTM carriers, proposing alternatives to overcome the problems that hamper the growth of the technology. These authors called attention to the fact that poisoning sources and membrane deactivation factors had not been properly analyzed in the published material.

### 3.8. The Poisonous Agents and the Lifetime

**Figure 11:**
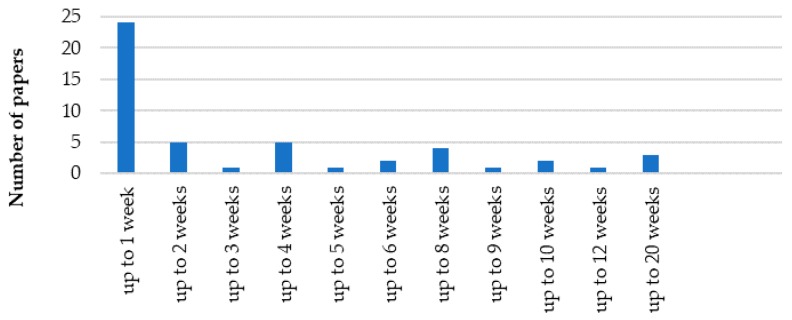
Lifetimes of membranes used for separation of gaseous paraffin/olefin mixtures in FTM processes.

It is important to emphasize that very few works analyzed the influence of contaminants [59] and the long-term performances of the separation modules [254], as shown in Figure 11. As a matter of fact, the presence of contaminants can exert strong negative effects on the performances of commercial scale membrane separation processes [59], which indicates that published papers regard the characterization of ideal separations and neglect the complexity of gaseous mixtures processed industrially. The poisonous agents identified in this work were acetylene and sulfur [125,207], reduction [90] and silver deposition [158], ketone [255], membrane dehydration [161], olefins and hydrogen [207].

Table 13 and Figure 11 present the membrane lifetimes reported in several papers, showing very short lifetimes in most cases. Although it is true that most published material regard lab-scale operations, this certainly is an indication of frequent process interruptions, changes of membrane modules and regeneration of separation units, rendering the process operation less efficient and more expensive [17]. Among the analyzed studies, the silver based CAF (amorphous fluoropolymer) membrane developed by Compact Membrane Systems (CMS) seemed to present superior performances in terms of operation lifetimes and selectivities for propylene/propane separations (although, according to the authors, similar results could be obtained with ethylene/ethane separations) [207]. This study reported selectivities of 50 and permeabilities of 200 GPU of propylene over a period of 300 d in lab-scale operations. Besides, the membrane was shown to be stable in presence of hydrogen sulfide, acetylene and hydrogen, although membrane humidification was needed to improve the process performance and stability.

### 3.9. The Layouts and Operation Conditions

**Figure 12:**
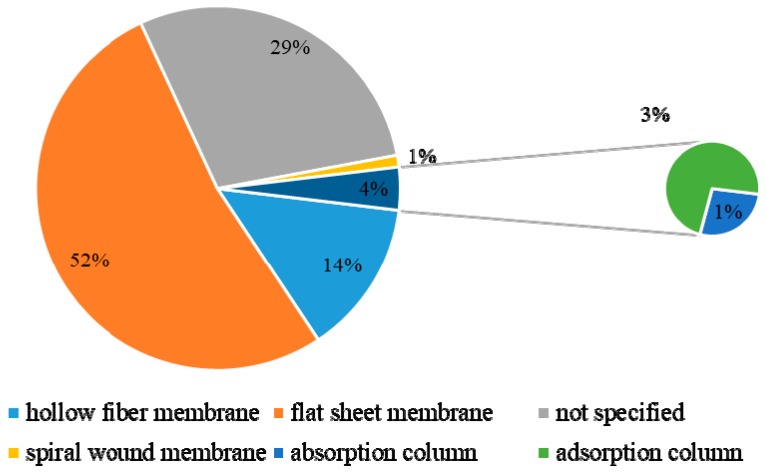
Distribution of layouts used to perform separations of gaseous paraffin/olefin mixtures.

**Figure 13:**
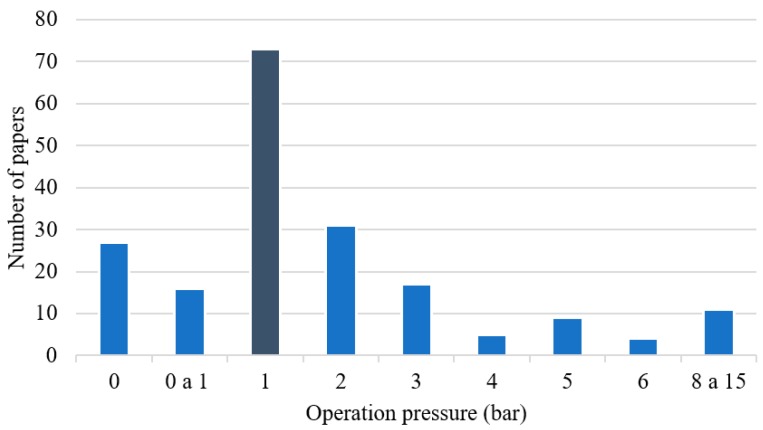
Pressures used to perform separations of gaseous paraffin/olefin mixtures.

**Figure 14:**
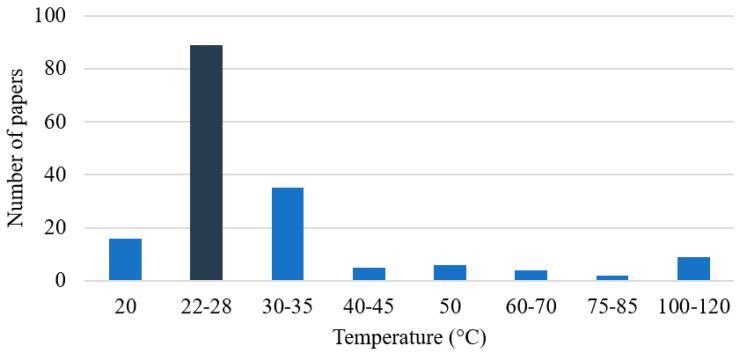
Temperatures used to perform separations of gaseous paraffin/olefin mixtures.

Figure 12 shows the equipment layouts used to perform membrane separations in different publications. The commonest membrane layouts employed in the analyzed articles were flat sheets (52% of the total number of papers), although it is important to note that the layout was not specified in 29% of the papers. Given the fact that most of these papers conducted separation tests in lab-scale units, the number of flat sheets is probably larger than the shown value. Only 14% of the documents employed hollow fiber membranes, while just 1% of the papers reported the use of spiral wound membranes. These numbers reinforce the fact that the vast majority of the investigations made use of small lab-scale setups for characterization of membrane performances and did not analyze the performances of larger commercial scale units. Therefore, once more it can be observed that the published material suggests that the degree of technological maturity of these processes is small and that research is concentrated on development and characterization of membrane materials, not on the implementation of actual commercial separation equipment. Moreover, the use of soft operating conditions (mainly 1 bar of pressure and ambient temperature) in most published documents indicates the lab scale characterization, as reported Figure 13 and Figure 14.

## 4. Conclusions

The present study surveyed the research on paraffin/olefin separations using membranes during the 1960–2019 period. Relevant information related to annual publication distribution, journals, main countries and institutions was analyzed. The first document analyzed was published in 1961 and the researches until 2010 were very limited. The journal that published more documents was Journal of Membrane Science, followed by Industrial and Engineering Chemistry Research and Separation and Purification Technology, publishing together almost 50% of the analyzed papers. The USA was the most productive country followed by South Korea and Iran. The second position attained by South Korea must be highlighted, since the Korea Institute of Science and Technology ranked in first among the top five most productive institutions.

The use of membranes for paraffin/olefin separation has not been successful for commercial applications yet. Zeolites, CMS and MOFs are new types of materials that have been studied more deeply since 2010 in order to overcome stability, selectivity and permeability issues encountered in other types of membranes. However, studies are still in lab scale. Facilitated transport separation using Ag as carrier was clearly the most relevant application and much research effort has been devoted to this topic.

Ethylene/ethane separations have been investigated to less extension than propylene/propane separations. Nonetheless, the separation of a great diversity of mixtures of organic compounds has been proposed. Flat sheet membranes dominate most studies and operating conditions are typical of lab-scale operations, suggesting that the use of membranes for paraffin/olefin separations still constitutes an immature field that has not been established industrially. This lack of technological maturity can be supported by many aspects. First, despite the fact that this issue has been discussed since the 1960s, the number of papers related to this theme is relatively small and the rate of publications in this field has not increased much since then. Besides, the types of materials reported for manufacture of the membranes is huge, indicating that consensus regarding the materials that are best suited for the analyzed applications has yet to be reached. Additionally, most membranes used for paraffin/olefin separations present short lifetimes, usually shorter than 2 weeks. This scenario possibly explains why reported membrane areas, flows, temperatures and feed pressures were obtained in laboratory scale and using ideal gas mixtures.

In spite of the current scenario, one cannot deny the many significant improvements achieved in this field. For instance, development of porous membranes with well-defined pore size distributions, including CMSs, zeolites, PIMs and MOFs, can overcome the inherent separation limits of dense polymer membranes. Also, some studies showed that conventional membranes based on solution–diffusion mechanisms are inefficient to produce high-purity olefin streams and that facilitated transport membranes (particularly the ones that contain silver-based carriers) constitute promising candidates to achieve high selectivity and permeability. Finally, based in the bibliometric analysis presented in the present study, it seems plausible to affirm that membranes that include the use of silver as a carrier (facilitated transport membranes and MOFs) seem to constitute the most promising technologies so far. However, these membranes are very sensitive and may be deactivated in the presence of H_2_S, sulfur, acetylene, olefins and by the reduction of the metal used as the olefin carrier, which demands additional and detailed studies about the effects of poisoning and membrane operation on the performance and stability of membrane separation units.

## Figures and Tables

**Table 1:** Characteristics of membrane systems used most often to separate olefin/paraffin streams.

Membrane Type	Definition and Characteristics	Drawbacks
CMS	Carbon molecular sieves constitute a class of amorphous carbon materials produced through the pyrolysis of microporous polymer precursors [8,59,62]. Although the surface area is relatively small, the characteristic pore sizes are small with narrow size distributions, enabling the separation at molecular level based on the size and shape of the molecules [8,63].	The pore diameters can be significantly different from characteristic sizes of molecules that must be separated. CMS materials can be fragile and it may be difficult to scale-up the production process [59,64].
Polymer	Polymer membranes can be casted with different thicknesses and porosities (PIMs) [2,58]. Carriers can be easily added to allow for; facilitated transport [8,65].	Polymer films can present low gas permeabilities and selectivities [8] and are subject to swelling, plasticization, and heterogeneous structure and porosity [66]. Carriers can be subject to deactivation by poisonous agents [59].
Zeolite	Zeolites are hydrated aluminosilicate materials, which possess outstanding ion-exchange and sorption properties [8,67]. Separation is based on pore sizes and polarity, which can be uniform [66] and are controllable [8]. Zeolites present higher thermal and chemical stabilities than polymers, large surface areas, high selectivities and high permeabilities [8,68]	Preparation conditions can be aggressive, with combination of high temperatures, high pressures and extreme pH values. The ranges of pore sizes can be narrow, adhesion properties onto different substrates can be poor and the production costs can be high [66,69].
MOF	Metal organic frameworks are hybrid materials constituted by metallic nodes, which are linked to each other through organic bridges, leading to functional porous structures [66,70].	The manufacture of continuous MOF layers can be difficult and the produced films can be very fragile. Adhesion properties onto different substrates can be poor and the production costs can be high [66].
MMM	Mixed matrix membranes are hybrid materials produced through mixing of polymers and inorganic fillers, including activated carbon, carbon nanotubes, zeolites, silica, molecular sieves, and MOFs [66]. Consequently, the final membrane properties can be manipulated with high flexibility.	The matrix and fillers must be compatible and filler aggregation and sedimentation must be prevented during membrane preparation [8].

**Table 2:** Usual mechanisms of olefin/paraffin separation through membranes.

Separation Mechanisms	Membrane Material	Permeation Mechanisms	Drawbacks
Solution-diffusion	Polymers	(1) Molecules adsorb and dissolve into the membrane material. (2) Molecules diffuse through the membrane, driven by pressure, temperature or concentration gradients. (3) Molecules desorb into the bulk stream in the permeate side [8].	Gas solubility in conventional polymer membranes is closely related to compressibility [8]. Discrimination of olefin/paraffin pairs is not effective [59].
Interaction between olefin and membrane	Zeolites, polymers, MOF, MMM, ionic liquids, adsorbents, absorbents	(1) Carriers can form complexes with gaseous components and allow the facilitated transport [59]. (2) The adsorption step can be followed by stepwise thermal regeneration and desorption [1].	Carriers are subject to deactivation by poisonous agents [59] and can be very expensive [71].
Molecular sieving	MOF, CMS, zeolites	(1) Molecules are separated due to different molecular sizes and shapes (geometrical selectivity) [8].	The pore diameters can be significantly different from characteristic sizes of molecules that must be separated. It may be difficult to scale-up the production process [59].

**Table 3:** Information categories investigated in the present study.

#	Category	#	Category	#	Category
1	Institution	6	Feed composition	11	Separated gases
2	Country	7	Selectivity or separation factor	12	Type of material
3	Journal	8	Permeability	13	Metal carrier
4	Year of publication	9	Operation temperature/°C	14	Layout
5	Number of citations	10	Operation pressure/bar	15	Lifetime

**Table 4:** Distribution of papers in scientific journals in the field of membrane paraffin/olefin gas separations.

Ranking	Journal	IF	NP	Percentage (%)
1	Journal of Membrane Science	6.03	101	34%
2	Industrial and Engineering Chemistry Research	2.84	32	11%
3	Separation and Purification Technology	3.35	14	5%
4	Microporous and Mesoporous Materials	3.61	5	2%
5	Journal of the American Chemical Society	13.85	5	2%
6	Chemical Communications	6.31	5	2%
7	Separation Science and Technology	1.10	5	2%
8	Chemical Engineering Science	2.89	5	2%

NP: Number of Publications; IF: Impact Factor.

**Table 5:** Distribution of papers in the 9 most productive countries in the field of membrane paraffin/olefin gas separations (with more than 10 papers).

Ranking	Country	Total Publications	Percentage (%)
1	USA	63	21%
2	South Korea	46	16%
3	Iran	27	9%
4	China	26	9%
5	Japan	20	7%
6	Netherlands	18	6%
7	Malaysia	13	5%
8	Germany	12	4%
9	Spain	10	4%

**Table 6:** Distribution of patents in the 3 most productive countries in the field of membrane paraffin/olefin gas separations (with more than 10 patents).

Ranking	Country	Percentage (%)
1	USA	56
2	Korea	21
3	France	12

**Table 7:** Distribution of papers in the 4 most productive institutions in the field of membrane paraffin/olefin gas separations (with more than 10 papers).

Ranking	Institutions	Documents	Percentage (%)
1	Korea Institute of Science and Technology (South Korea)	21	7
2	Amirkabir University of Technology (Iran)	12	4
2	Georgia Institute of Technology (USA)	12	4
3	University of Twente (The Nerthelands)	10	3

**Table 8:** Distribution of patents in the 6 most productive institutions in the field of membrane paraffin/olefin gas separations.

Ranking	Institution	Countries	Percentage (%)
1	ExxonMobil Research and Engineering Company	USA	9
1	UOP LLC	USA	9
1	Institut Français du Petrole	France	9
1	Industry-University Cooperation Foundation Hanyang University	Korea	9
1	Korea Institute of Science and Technology	Korea	9
2	Membrane Technology and Research, Inc.	USA	6

**Table 9:** The most cited papers in the field of membrane paraffin/olefin gas separations.

Ranking	Paper	Separated Gases	Type of Membrane (Name)	Citations	Ref.
**1**	Hydrocarbon Separations in a Metal-Organic Framework with Open Iron(II) Coordination Sites Authors: Bloch, E.D., Queen, W.L., Krishna, R., Zadrozny, J.M., Brown, C.M., Long, J.R. Source: Science (2012)	Ethylene/Ethane Propane/Propane	MOF (Fe2(dobdc))	1008	[101]
**2**	Pushing the limits on possibilities for large scale gas separation: which strategies? Authors: Koros, W.J., Mahajan, R. Source: Journal of Membrane Science (2000)	Olefin/Paraffin Others	Various (Review)	829	[102]
**3**	Gas solubility, diffusivity and permeability in poly(ethylene oxide) Authors: Lin, H., Freeman, B.D. Source: Journal of Membrane Science (2004)	Ethylene/Ethane Propylene/Propane Others	Polymer (PEO)	627	[103]
**4**	Olefin/Paraffin Separation Technology: A Review Author: Eldridge, R.B. Source: Industrial and Engineering Chemistry Research (1993)	Olefin/Paraffin	Various (Review)	580	[1]
**5**	Application of membrane separation processes in petrochemical industry: a review Authors: Ravanchi, M.T., Kaghazchi, T., Kargari, A. Source: Desalination (2009)	Propylene/Propane	Polymer (6FDA-DDBT)	487	[53]
**6**	Title: Zeolitic Imidazolate Frameworks for Kinetic Separation of Propane and Propene Author(s): Li, K., Olson, D.H., Seidel, J., Emge, T.J., Gong, H., Zeng, H., Li, J. Source: Journal of the American Chemical Society (2009)	Propylene/Propane	MOF (ZIF-8)	466	[104]
**7**	Title: Ethane/Ethene Separation Turned on Its Head: Selective Ethane Adsorption on the Metal-Organic Framework ZIF-7 through a Gate-Opening Mechanism. Author(s): Gücüyener, C., Bergh, J.V.D., Gascon, J., Kapteijn, F. Source: Journal of the American Chemical Society (2010)	Ethylene/Ethane	MOF (ZIF-7)	408	[77]
**8**	Title: Olefin/Paraffin Separations by Reactive Absorption: A Review Author(s): Safarik, D.J., Eldridge, R.B. Source: Industrial and Engineering Chemistry Research (1998)	Olefin/Paraffin	Absorbent (Review)	312	[105]

**Table 10:** Characteristic FTM parameters for different carriers in membrane paraffin/olefin separations of gaseous streams.

Separated Gases	Type of Material	Name of the Material	Carrier	Selectivity or Sep Factor	Ref.
ethylene/ethane	FT/Hybrid	not specified	Ag	NS	[125]
ethylene/ethane	FT/Hybrid	not specified	Ag^+^	SF 65 ethylene/ethane	[126]
ethylene/ethane	FT/Hybrid	Chitosan/Ag (Imtex)	Ag^+^	SF 100 ethylene/ethane	[127]
ethylene/ethane	FT/Hybrid	5A zeolite	Ag^+^	S 27.4 ethylene	[128]
ethylene/ethane	FT/Hybrid	Fe2(dobdc)	Ag^+^	S 13.6 ethylene	[128]
ethylene/ethane	FT/Liquid	Fluoropore FP-010/AgNO_3_ (Sumitomo)	Ag^+^	SF 460 ethylene/ethane	[90]
ethylene/ethane	FT/Liquid	polysulfone	Ag^+^	SF 420 ethylene/ethane	[129]
ethylene/ethane	FT/Liquid	PEO/PBT/AgNO_3_	Ag^+^	SF 165 ethylene/ethane	[130]
ethylene/ethane	FT/Liquid	EPDM-SPEEK	Ag^+^	SF 2700 ethylene/ethane	[131]
ethylene/ethane	FT/Liquid	[4-mebupy]BF_4_	Ag^+^	S 3 ethylene	[132]
ethylene/ethane	FT/Liquid	Cu SILM supported PVDF	Cu	S 11.8	[133]
ethylene/ethane	FT/Liquid	PIL/40IL-Ag^+^ 1.25 M	Ag^+^	S 7.24 etylene	[134]
ethylene/ethane	FT/Liquid	ZnCl_2_/[BMIM][Cl]	Zn IL	S 178	[135]
ethylene/ethane	FT/Liquid	CuCl/ChCl-EG-based SLMs	Cu	S 12.5	[136]
ethylene/ethane	FT/Liquid	CuCl/DESs-SLMs	IL	SF 20 ethylene/ethane	[135]
ethylene/ethane	FT/Liquid	DESs-SLMs	Ag^+^	S 50 -100 ethylene	[111]
ethylene/ethane	FT/Polymer	Nafion N-117	Ag^+^	SF 540 ethylene/ethane	[137]
ethylene/ethane	FT/Polymer	AgBF_4_/PVP	Ag^+^	SF 2.3 ethylene/ethane	[138]
ethylene/ethane	FT/Polymer	AgBF_4_/PEO	Ag^+^	SF 240 ethylene/ethane	[139]
ethylene/ethane	FT/Polymer	Pebax® 4011 and Pebax® 2533 (Atofina)	Ag^+^	NS	[140]
ethylene/ethane	FT/Polymer	AgNO_3_/polyethersulfone (Daicel)	Ag^+^	SF 1100 ethylene/ethane	[141]
ethylene/ethane	FT/Polymer	PA 1 2-PTMO/AgBF_4_	Ag^+^	SF 20 ethylene/ethane	[142]
ethylene/Ethane	FT/Polymer	POZ/AgBF_4_	Ag^+^	SF 5 ethylene/ethane	[143]
ethylene/ethane	FT/Polymer	EPDM	Ag^+^	SF 72.5 ethylene/ethene	[136]
ethylene/ethane	FT/Polymer	AgNO_3_/polyethersulfone (Daicel)	Ag^+^	SF 374 ethylene/ethane	[144]
ethylene/ethane	FT/Polymer	PebaxTM 2533/AgBF_4_	Ag^+^	NS	[145]
ethylene/ethane	FT/Polymer	3c	Ag^+^	SF 115 ethylene/ethane	[146]
ethylene/ethane	FT/Polymer	SiO_2_ Poly(sodium acrylate) Ag^+^	Ag^+^	SF 94 ethylene/ethane	[147]
ethylene/ethane	FT/Polymer	Pebax® 2533/AgBF_4_ (Arkema)	Ag^+^	SF 55 ethylene/ethane	[115]
ethylene/ethane	FT/Polymer	28% PVDF/72% triacetin/AgNO_3_	Ag^+^	NS	[129]
ethylene/ethane	FT/Polymer	Psf/AgNO_3_	Ag^+^	NS	[148]
ethylene/ethane	FT/Polymer	PSf/PTMSP	Ag^+^	NS	[149]
ethylene/ethane	FT/Polymer	AgBF_4_-PVMK membrane	Ag^+^	ethylene/ethane	[150]
ethylene/ethane	FT/Polymer	PEO-AgBF_4_	Ag^+^	NS	[151]
propylene/propane	FT/Hybrid	Ag/SBA-15	Ag^+^	S 10 propylene	[152]
propylene/propane	FT/Hybrid	Ag/c-Al_2_O_3_	Ag^+^	S 1.2 propane	[153]
propylene/propane	FT/Hybrid	POZ/AgNO_3_/SiO_2_ (fumed silica nanoparticles) (1:1:0.1)	Ag^+^	S 90.0 propylene/propane	[154]
propylene/propane	FT/Hybrid	POZ/AgNO_3_/BMIM^+^NO_3_^−^	Ag^+^	S 32.0 propylene/propane	[155]
propylene/propane	FT/Hybrid	POZ/AgNO_3_/BMIM^+^BF_4_^−^	Ag^+^	S 31.8 propylene/propane	[155]
propylene/propane	FT/Hybrid	POZ/AgNO_3_/BMIM^+^CF_3_SO_3_^−^	Ag^+^	S 33.2 propylene/propane	[155]
propylene/propane	FT/Hybrid	PVP/Nano Au (Seahan)	Au	S 22 propylene	[96]
propylene/propane	FT/Hybrid	POZ	Ag^+^	SF 20–22.5 propylene/propane	[156]
propylene/propane	FT/Hybrid	PVDF-HFP/BMImBF_4_^−^Ag^+^	Ag^+^	S 700 propane	[157]
propylene/propane	FT/Hybrid	AgNO_3_/Al_2_O_3_	Ag^+^	NS	[158]
propylene/propane	FT/Hybrid	MICRODYN MD020 TP 2N	Ag^+^	NS	[159]
propylene/propane	FT/Hybrid	TiO_2_-PEO-AgBF_4_	Ag^+^	S 19 propylene/propane	[160]
propylene/propane	FT/Hybrid	Permylene (Imtex)	Ag^+^	NS	[161]
propylene/propane	FT/Hybrid	PHMEP-g-PEGBEM/AgBF_4_/MgO-NS	Ag^+^	SF 12.9 propylene/ propane	[162]
propylene/propane	FT/Liquid	POZ/AgNO_3_/BMIM^+^BF_4_^−^	Ag^+^	SF 31.8 propylene/propane	[163]
propylene/propane	FT/Liquid	POZ/AgNO_3_/BMIM^+^NO_3_^−^	Ag^+^	SF 32 propylene/propane	[163]
propylene/propane	FT/Liquid	zirconia/AgNO_3_	Ag^+^	SF 20 propylene/propane	[164]
propylene/propane	FT/Liquid	TEG/AgBF_4_	Ag^+^	SF 60 propylene/propane	[165]
propylene/propane	FT/Liquid	AgBF_4_	Ag^+^	S 4.5 propylene	[166]
propylene/propane	FT/Liquid	PVDF/AgNO_3_	Ag^+^	SF 474 propylene/propane	[167]
propylene/propane	FT/Liquid	BMIM^+^BF_4_^−^/Ag	Ag^+^	SF 17 propylene/propane	[168]
propylene/propane	FT/Liquid	Ag-BMImBF_4_	Ag^+^	NS	[169]
propylene/propane	FT/Liquid	AgNO_3_/PVDF (Millipore)	Ag^+^	NS	[170]
propylene/propane	FT/Liquid	BMIM^+^BF_4_^−^	Cu	SF 5.2 propylene/propane	[93]
propylene/propane	FT/Liquid	PVDF/AgNO_3_	Ag^+^	SF 480 propylene/propane	[171]
propylene/propane	FT/Liquid	PVDF/AgNO_3_	Ag^+^	SF 490 propylene/propane	[172]
propylene/propane	FT/Liquid	[Ag(propene)x][Tf2N]	Ag^+^	SF 3 propane/propene	[173]
propylene/propane	FT/Liquid	RTILs	Ag^+^	SF 100 propylene/propane	[174]
propylene/propane	FT/Liquid	PVDF/AgNO_3_	Ag^+^	SF 270 propylene/propane	[175]
propylene/propane	FT/Liquid	BMImBF_4_	Ag^+^	SF 20 propylene/propane	[176]
propylene/propane	FT/Liquid	MOIM^+^NO_3_^−^	IL	SF 2.8 propylene/propane	[99]
propylene/propane	FT/Liquid	BMIM^+^BF_4_^−^	IL	SF 2.3 propylene/propane	[99]
propylene/propane	FT/Liquid	AgNO_3_ in hollow fiber membrane	Ag^+^	75% propylene removal	[177]
propylene/propane	FT/Liquid	(Emim,Ag)[BF_4_]^−^PICPM^+^PF_6_^−^	Ag^+^	SF 7 propylene/propane	[178]
propylene/propane	FT/Liquid	(Emim,Ag)[Tf2N]^−^PICPM^+^Tf2N^−^	Ag^+^	SF 7 propylene/propane	[178]
propylene/propane	FT/Liquid	(Emim,Ag)[Tf2N]-12HSA	Ag^+^	SF 7 propylene/propane	[178]
propylene/propane	FT/Liquid	MOIM^+^BF_4_^−^/Cu	Cu	SF 2 propylene/propane	[179]
propylene/propane	FT/Liquid	PVDF/AgNO_3_	Ag^+^	SF 473.86 propylene/propane	[118]
propylene/propane	FT/Liquid	NMP	Ag^+^	S 4.5 propylene	[180]
propylene/propane	FT/Polymer	PVA/AgSbF_6_	Ag^+^	S 125 propylene	[181]
propylene/propane	FT/Polymer	PVDFHFP/BMImBF_4_/AgBF_4_	Ag^+^	NS	[182]
propylene/propane	FT/Polymer	PE-g-AA-Ag^+^	Cu	SF 21 propylene/propane	[60]
propylene/propane	FT/Polymer	PPO	Ag^+^	SF 5.33 propylene/propane	[183]
propylene/propane	FT/Polymer	Cu/PVP	Cu	SF 10 propylene/propane	[184]
propylene/propane	FT/Polymer	AgNO_3_/PEG/Psf	Ag^+^	SF 250 propylene/propane	[185]
propylene/propane	FT/Polymer	AgBF_4_-PVP	Ag^+^	SF 140 propylene/propane	[124]
propylene/propane	FT/Polymer	POZ	Ag^+^	SF 280 proylene/propane	[186]
propylene/propane	FT/Polymer	PEO	Ag^+^	NS	[187]
propylene/propane	FT/Polymer	AgBF_4_-PVP	Ag^+^	SF 140 propylene/propane	[188]
propylene/propane	FT/Polymer	AgBF_4_-POZ	Ag^+^	SF 130 propylene/propane	[188]
propylene/propane	FT/Polymer	PVP/AgBF_4_	Ag^+^	NS	[189]
propylene/propane	FT/Polymer	PVP/AgBF_4_	Ag^+^	SF 60 propylene/propane	[190]
propylene/propane	FT/Polymer	PVP/AgNO_3_/Ppy	Ag^+^	NS	[191]
propylene/propane	FT/Polymer	POZ	Ag^+^	SF 5 propylene/propane	[192]
propylene/propane	FT/Polymer	PEP/AgBF_4_	Ag^+^	SF 55 propylene/propane	[94]
propylene/propane	FT/Polymer	PDMS/AgBF_4_	Ag^+^	SF 200 propylene/propane	[193]
propylene/propane	FT/Polymer	PHMV	Ag^+^	S 336 propylene	[194]
propylene/propane	FT/Polymer	POZ	Ag^+^	SF 65 propylene/propane	[195]
propylene/propane	FT/Polymer	PVP/silver salts	Ag^+^	NS	[196]
propylene/propane	FT/Polymer	POZ/AgBF_4_	Ag^+^	SF 45 propylene/propane	[197]
propylene/propane	FT/Polymer	6FDA–4MPD/DABA	Ag^+^	S 10 propylene/propane	[198]
propylene/propane	FT/Polymer	BMIM^+^BF_4_	Ag^+^	SF 17 propylene/propane	[95]
propylene/propane	FT/Polymer	SBS/0.5Ag	Ag^+^	S 80 propylene/propane	[199]
propylene/propane	FT/Polymer	Ag–sugar/BMIM^+^BF_4_^−^ (0.05/1)	Ag^+^	SF 12.9 propylene/propane	[200]
propylene/propane	FT/Polymer	PVC-g-P4VP	Ag^+^	S 6 propylene	[201]
propylene/propane	FT/Polymer	PEI/Pebax2533/AgBF_4_	Ag^+^	SF 1000 propylene/propane	[202]
propylene/propane	FT/Polymer	PU/AgCF_3_SO_3_ (BASF )	Ag^+^	S 10 propylene	[203]
propylene/propane	FT/Polymer	PTFE (Mencor)	Ag^+^	60% propylene	[121]
propylene/propane	FT/Polymer	PP/AgBF_4_	Ag^+^	NS	[204]
propylene/propane	FT/Polymer	polymer membranes with inorganic nanoparticles uniformly dispersed	Zn	SF 18.08 propylene/propane	[205]
propylene/propane	FT/Polymer	Pebax® 1657/AgBF_4_ (Atofina)	Ag^+^	SF 20.4 propylene/propane	[206]
propylene/propane	FT/Polymer	poly(vinylalcohol)/AgBF_4_/Al(NO_3_)_3_	Ag^+^	SF 17 propylene/propane	[98]
propylene/propane	FT/Polymer	(PVA)/AgBF_4_/Al(NO_3_)_3_	Ag^+^	NS	[98]
propylene/propane	FT/Polymer	PVP/AgBF_4_/Al(NO_3_)_3_/Ag_2_O	Ag^+^	SF 21.7 propylene/propane	[100]
propylene/propane	FT/Polymer	CAF (CMS)	Ag^+^	SF 50 propylene/propane	[207]
propylene/propane	FT/Polymer	SBS/Cu@MIL-101(Cr) MMM	Cu	S 2 propylene	[208]
propylene/propane	FT/Polymer	PE-g-AA-Ag^+^	Ag^+^	S 5 propane	[209]
propylene/propane	FT/Polymer	PE-g-AA-Cu^+^	Cu^+^	S 2.2 propane	[209]
propylene/propane	FT/Polymer	PE-g-AA-Cu^2+^	Cu^2+^	S 1.7 propane	[209]
propylene/propane	FT/Polymer	PEO-AgBF_4_	Ag^+^	NS	[151]

The separation factor (SF) of the gas pairs may be defined as the quotient between the molar ratios of the components in the permeate side divided by the quotient between the molar ratios of the components in the feed side. The ideal selectivity (S) is calculated as the ratio between the permeances of the individual components. NS stands for not specified.

**Table 11:** Reference values reported for gas separations with help of CMS membranes.

Separated Gases	Name of the Material	Selectivity or Sep Factor	Permeability or Permeance	Temp (K)	Pressure (bar)	Ref.
ethylene/ethane	Carbonized BPDA-pp’ODA Polyimide	SF 5 ethylene/ethane	P 1 ethylene (×10^−8^ mol m^−2^ s^−1^ Pa^−1^)	373	1.013	[81]
ethylene/ethane	Matrimid® 5218 (Huntsman)	S 12 ethane	P 14.4 (barrer)	308	NS	[215]
ethylene/ethane	Matrimid® 5218 (Huntsman)	S 12 ethylene/ethane	P 14–15 ethylene (barrer)	308	3.447	[216]
ethylene/ethane	Matrimid	SF 60 ethylene/ethane	P 4.8 × 10^−7^ ethylene; P 1.6 × 10^−9^ ethane (mol·Pa^−1^·m^−2^·s^−1^)	NS	NS	[217]
ethylene/ethane	6FDA/BPDA-DAM	SF >20	P 10 ethylene GPU	308	20.265	[218]
ethylene/ethane	PIM-6FDA-OH	SF 17.5 ethylene/ethane	P 10 ethylene (barrer)	308	20.265	[219]
ethylene/ethane	Matrimid and 6FDA/BPDA-DAM	NS	NS	308	8.04	[220]
ethylene/ethane	6FDA/BPDA-DAM	S 3.9 ethylene/ethane	P 15.9 ethylene; P 4.0 ethane (GPU)	298	5.15	[221]
propylene/propane	6FDA/BPDA–DDBT	S 22 propylene	P 26 GPU propylene	373	1.013	[84]
propylene/propane	NTDA-BAHFDS	S 42 propane	P 26 GPU propylene/propane	308	1.013	[86]
propylene/propane	AlPO-14	NS	NS	NS	NS	[222]
propylene/propane	6FDA/BPDA-DAM	S 20.5 propylene/propane	P 17.5 propylene; P 0.85 propane (GPU)	298	5.15	[221]
propylene/propane	CMS/g-Al2O3	SF 36 propylene/propane	P 9 GPU propylene	298	1.3–4	[223]
propylene/propane	6FDA	S 50–60 propylene	P 8 propylene/propane [×10^−9^ mol/(m^2^ s Pa)]	393	6.89	[224]
propylene/propane	CMS membranes synthesized on mesoporous g-alumina support	SF 31 propylene/propane	P 1.0 [× 10^−8^ mol m^−2^ s^−1^ Pa^−1^]	298	3.1	[63]
propylene/propane	BPDA-DDBT/DABA	SF 13 propylene/propane	P 50 GPU propane	373	1.013	[82]

**Table 12:** Reference values reported for gas separations using zeolite and MOF membranes.

Separated Gases	Name of the Material	Selectivity or Sep Factor	Permeability or Permeance	Temp (K)	Pressure (bar)	Ref.
ethylene/ethane	CuCl-modified tubular γ-Al_2_O_3_ membrane	SF 1.4 ethylene/ethane	NS	333	2.026	[226]
ethylene/ethane	CuCl/NaX	NS	NS	358	2	[227]
ethylene/ethane	Na-ETS-10	S 5 ethylene	NS	298	1.013	[228]
ethylene/ethane	AgA and AgX	NS	NS	303	1.013	[229]
ethylene/ethane	ZIF-4 and ZIF-zni	NS	NS	293	NS	[230]
ethylene/ethane	ZIF-4	SF 1.71 ethane/ethylene	NS	293	up to 12	[231]
ethylene/ethane	Ag-X	S 15.9 ethylene	P 9.04 ¹	303	NS	[232]
ethylene/ethane	6FDA-DAM:DABA	SF 9 ethylene/ethane	P 90 ethylene (barrer)	308	3.44	[233]
ethylene/ethane	ZIF-7	NS	NS	NS	0	[77]
ethylene/ethane	ZIF-8	S 2.8 ethylene	P 1.5 ethylene ¹	298	1	[78]
ethylene/ethane	Cu3BTC2	SF 7.1 ethylene/ethane	P 17 ¹	423	5	[234]
ethylene/ethane	Cu3BTC2	SF 7.1 ethylene/ethane	P 17¹	423	5	[234]
ethylene/ethane	IRMOF-8	S 1.43 Ethane/Ethylene	NS	318	8	[235]
ethylene/ethane	MIL-101	SF 16.5 ethylene/ethane	NS	303	1	[236]
ethylene/ethane	MIL-100	111 ethylene/ethane	NS	298	0.01	[225]
ethylene/ethane	M–MOF-74	SF 10 ethylyne/ethane	NS	318	1	[237]
ethylene/ethane	Mg2(dhtp)	S 1.4 ethylene/ethane	NS	293	0.015	[238]
ethylene/ethane	Co2(dhtp)	S 1.7 ethylene/ethane	NS	293	0.015	[238]
ethylene/ethane	ZIF-8	S 0.48 ethylene/ethane	NS	293	0.015	[238]
ethylene/ethane	Fe2(dobdc)	NS	NS	318	NS	[101]
ethylene/ethane	CuBTC	NS	NS	303; 373	0.01–5	[239]
ethylene/ethane	ZIF-71	SF 1.84 propane/propylene	NS	293	1	[240]
propylene/propane	Mg2(dhtp)	S 1.7 propylene/propane	NS	293	0.015	[238]
propylene/propane	Co2(dhtp)	S 2.9 propylene/propane	NS	293	0.015	[238]
propylene/propane	ZIF-8	S 0.7 propylene/propane	NS	293	0.015	[238]
propylene/propane	Fe2(dobdc)	NS	NS	318	NS	[101]
propylene/propane	CuBTC	NS	NS	303; 373	0.01 - 5	[239]
propylene/propane	ZIF-8	NS	NS	NS	1	[241]
propylene/propane	Basolite® C300 (BASF)	NS	NS	323–373	5	[79]
propylene/propane	6FDA-Durene/DABAco-polyimides ZIF-8	SF 27.38 propylene/propane	NS	308	10.13	[242]
propylene/propane	NbOFFIVE-1-Ni (KAUST-7)	NS	NS	298	1	[243]
propylene/propane	ZIF-9	SF 1.39 ethane/ethylene	NS	293	1	[240]
propylene/propane	Zr-fum-fcu-MOF	NS	NS	328	NS	[80]
propylene/propane	MIL-100	70 propylene/propane	NS	298	0.01	[225]

¹ [×10^−8^ mol m^−2^ s^−1^ Pa^−1^].

**Table 13:** Lifetimes of membranes used for separation of gaseous paraffin/olefin mixtures in FTM processes.

Separated Gases	Name of the Material	Carrier	Temp (K)	Pressure (bar)	Lifetime	Ref.
1-butene/n-butane	ILMs in PVDF substrates	Ag^+^	NS	0.14	at least 600 h	[256]
ethylene/ethane	Fluoropore FP-010/AgNO_3_	Ag^+^	298	1.01	at least 100 h	[90]
ethylene/ethane	EPDM-SPEEK	Ag^+^	298	3	at least 1680 h	[131]
ethylene/ethane	ZnCl_2_/[BMIM][Cl]	Cu	298	1.1	150 h	[257]
ethylene/ethane	AgBF_4_/PEO	Ag^+^	296	1.72	at least 16 h	[139]
ethylene/ethane	AgNO_3_/polyethersulfone	Ag^+^	298	0.09	1440 h	[141]
ethylene/ethane	PA 12-PTMO/AgBF_4_	Ag^+^	295	3.44	72 h	[142]
ethylene/ethane	EPDM	Ag^+^	298	3	over 3360 h	[136]
ethylene/ethane	AgNO_3_/polyethersulfone	Ag^+^	298	2	504 h	[144]
ethylene/ethane	SiO_2_ Poly(sodium acrylate) Ag^+^	Ag^+^	373	2	at least 5 h	[147]
ethylene/ethane	Pebax® 2533/AgBF_4_	Ag^+^	296	3.44	7 days	[115]
ethylene/ethane	Psf/AgNO_3_	Ag^+^	NS	1	1440 h	[148]
ethylene/ethane	PEO-AgBF_4_	Ag^+^	296	7.9	at least 20 h	[151]
i-butene/i-butane	(PTMSP-g-AA-Ag^+^)	Ag^+^	298	NS	at least 1008 h	[258]
isoprene/n-pentane	SPEEK-AgNO_3_	Ag^+^	333	101.325	100 h	[259]
pentene/pentane	Select	Ag^+^	298	1.013	48 h	[260]
propylene/propane	POZ/AgNO_3_/SiO_2_	Ag^+^	293	2.75	160 h	[154]
propylene/propane	PVP/Nano Au	Au	298	1.013	2 days	[96]
propylene/propane	POZ	Ag^+^	293	2.75	14 days	[156]
propylene/propane	PVDF-HFP/BMImBF_4_^–^Ag^+^	Ag^+^	293–323	0.5–3	10 days	[157]
propylene/propane	AgNO_3_/Al_2_O_3_	Ag^+^	298	1	at least 4320 h	[158]
propylene/propane	TiO_2_-PEO-AgBF_4_	Ag^+^	298	1	less than 196 h	[160]
propylene/propane	Permylene	Ag^+^	298	5.56	over 1000 h	[161]
propylene/propane	POZ/P154AgNO_3_/BMIM^+^NO_3_^−^	Ag^+^	NS	NS	150 h	[163]
propylene/propane	TEG/AgBF_4_	Ag^+^	293–298	1.013	1440–2160 h	[165]
propylene/propane	PVDF/AgNO_3_	Ag^+^	298	1.2	2880 h	[167]
propylene/propane	BMIM^+^BF_4_^−^/Ag	Ag^+^	NS	2.75	at least 100 h	[168]
propylene/propane	AgNO_3_/PVDF	Ag^+^	298	1.2	3–4 weeks	[170]
propylene/propane	PVDF/AgNO_3_	Ag^+^	298	1.2	3–4 weeks	[175]
propylene/propane	PVDF/AgNO_3_	Ag^+^	NS	NS	2880 h	[118]
propylene/propane	NMP	Ag^+^	293	1.2–2.2	60 h	[180]
propylene/propane	Cu/PVP	Cu	298	1.38	168 h	[184]
propylene/propane	AgBF_4_-PVP	Ag^+^	NS	NS	at least 100 h	[124]
propylene/propane	POZ	Ag^+^	296	1.38	50 h	[186]
propylene/propane	AgBF_4_-PVP	Ag^+^	NS	NS	at least 100 h	[188]
propylene/propane	AgBF_4_-POZ	Ag^+^	NS	NS	at least 100 h	[188]
propylene/propane	PVP/AgBF_4_	Ag^+^	NS	2.76	720 h	[190]
propylene/propane	PEP/AgBF_4_	Ag^+^	293	2.758	150 h	[94]
propylene/propane	PDMS/AgBF_4_	Ag^+^	NS	1.38	at least 5.8 h	[193]
propylene/propane	PTFE	Ag^+^	298	1.2	2 months	[121]
propylene/propane	poly(vinylalcohol)/AgBF_4_/Al(NO_3_)_3_	Ag^+^	NS	3	145 h	[98]
propylene/propane	CAF (CMS)	Ag^+^	298	5.15	over 9 months	[207]
propylene/propane	PEO-AgBF_4_	Ag^+^	296	7.9	at least 20 h	[151]

## Supplementary Materials

The file of Supplementary Materials [261,262,263,264,265,266,267,268,269,270,271,272,273,274,275,276,277,278,279,280,281,282,283,284,285,286,287,288,289,290,291,292,293,294,295,296,297,298,299,300,301,302,303,304,305,306,307,308,309,310,311,312,313,314,315,316,317,318,319,320,321,322,323,324,325,326,327,328] are available online at https://www.mdpi.com/2077-0375/9/12/157/s1, Table S1. Distribution of membranes used for gas separations involving paraffins and/or olefins (background rated category), excluding the separation between paraffins and olefins; Table S2. Distribution of membranes and conditions used for paraffin/olefin separations; Table S3. Papers analyzed in the bibliometric study.
