# Supplementary material for: A Bibliometric Survey of Paraffin/Olefin Separation Using Membranes
Source: Membranes (Basel). 2019 Nov 26;9(12):157. doi: 10.3390/membranes9120157 (PMC6950670; doi:10.3390/membranes9120157)
Supplement: Supplementary file 1 [file membranes-09-00157-s001.pdf]

Table S1. Distribution of membranes used for gas separations involving paraffins and/or olefins (background rated category), excluding the separation between paraffins and olefins.

Separated Gases	Type of Membrane	Membrane (Supplier)	Carrier	Additional Information	Reference
CO_2/CH_4	Hybrid	MMMs	-	NS	[17]
CO_2/CH_4	Hybrid	10PEG/AC/SR/CA MMM	-	SF 14.12 CO_2/CH_4	[29]
CO_2/CH_4	Liquid	PES/CF_3SO_3 or Tf_2N	-	SF 24 CO_2/CH_4	[41]
CO_2/CH_4	FT/liquid	PVDF (Memcor Australia)	Ag^+	NS	[26]
CO_2/CH_4	MOF	UiO-66	-	NS	[261]
CO_2/CH_4	MOF	$Co_3(HCOO)_6$	Co	SF 15.95 CO_2/CH_4	[21]
CO_2/CH_4	MOF	Matrimids 5218/30%ZIF-8 (Huntsman)	-	SF 60 CO_2/CH_4	[22]
CO_2/CH_4	MOF	ZIF	-	NS	[38]
CO_2/CH_4	MOF	ZIF-8	-	NS	[28]
CO_2/CH_4	MOF	ZIF-8	-	182 CO_2/CH_4	[39]
CO_2/CH_4	Zeolite	Ba-SAPO-34	-	250 CO_2/CH_4	[16]
CO_2/CH_4	Zeolite	MTES	-	4.5 CO_2/CH_4	[13]
CO_2/CH_4	Zeolite	FAU-zeolite	-	4.48 CO_2/CH_4	[25]
CO_2/CH_4	Zeolite	DDR-type zeolite membrane	-	SF 200 CO_2/CH_4	[30]
CO_2/CH_4	Silica	BTESE-MTES hybrid silica	-	NS	[262]
CO_2/CH_4	Silica	HT-silica	-	42.7 CO_2/CH_4	[12]
CO_2/CH_4	CMS	CM-15%wt	-	SF 87.34 CO_2/CH_4	[27]
CO_2/CH_4	Polymer	PDMS/PSF	-	SF 4 CO_2/CH_4	[42]
CO_2/CH_4	Polymer	PSF/3% Zeolite-T	-	SF 3.37 CO_2/CH_4	[46]
CO_2/CH_4	Polymer	PC/PEG	-	SF 40.9 CO_2/CH_4	[18]
CO_2/CH_4	Polymer	Pebax-1657	-	28 CO_2/CH_4	[20]
CO_2/CH_4	Polymer	30% AC/PVA	-	100 CO_2/CH_4	[23]
CO_2/CH_4	Polymer	PSF Udel-3500/GO	--	25 CO_2/CH_4	[36]
CO_2/CH_4	Polymer	PEBAX/4.6 wt% FS	-	74.5 CO_2/CH_4	[43]
CO_2/CH_4	Polymer	PES/Pebax 1657	-	14 CO_2/CH_4	[14]
CO_2/CH_4	Polymer	Polyamide composite	-	SF 2.2 CO_2/CH_4	[9]
CO_2/CH_4	Polymer	6FDA-Durene/DABA	-	NS	[19]
CO_2/CH_4	Polymer	PES	-	SF 26.5 CO_2/CH_4	[35]
CO_2/CH_4	Polymer	PES/PU	-	SF 28 CO_2/CH_4	[34]
CO_2/CH_4	Polymer	PES/C15A2	-	SF 33.49 CO_2/CH_4	[10]
CO_2/CH_4	Polymer	COC/GO-SA	-	SF 12.5 CO_2/CH_4	[11]
CO_2/CH_4	Polymer	Udel 1700 PSF with DEG solvent	-	23.12 CO_2/CH_4	[32]
CO_2/CH_4	Polymer	PVAm/PVA	-	45 CO2/CH4	[45]
CO_2/CH_4	Polymer	y SSZ-13	-	300 CO_2/CH_4	[24]
CO_2/CH_4	Polymer	6FDA-TMPDA	-	SF 10 CO_2/CH_4	[44]
CO_2/CH_4	Polymer	PMDA-ODA-4,9	-	SF 31.55 CO_2/CH_4	[31]
H_2/CH_4	Polymer	COC/GO-SA	-	SF 498.8 H_2/CH_4	[11]
H_2/CH_4	Silica	BTESE-MTES hybrid silica	-	NS	[262]
H_2/CH_4	Zeolite	TEBAB	-	NS	[263]
He/CH_4	Polymer	ABS/PVAc/SiO2/SBR	-	SF 51.88 He/CH_4	[264]
H_2S/CH_4	CMS	graphene membranes	-	8.01 H_2S/ CH_4	[265]
H_2/propane	Zeolite	Al100P60Oz	-	SF 16 H_2/propane	[266]
H_2/propane	MOF	ZIF-8	-	SF 350 H_2/propane	[267]

propane/CO_2	Polymer	PDMS	-	NS	[268]
ethylene/air	FT/Liquid	Cu(I) solution	Cu	NS	[269]
ethylene/Argon	Polymer	VaporSep (MTR)	-	NS	[270]
propylene/nitrogen	Zeolite	ultra-thin MFI (0.5 μm)	-	SF 43 propylene at 296K	[271]
ethylene/nitrogen	Zeolite	ultra-thin MFI (0.5 μm)	-	SF 6 ethylene at 277K	[271]
i-butene/n-butene	FT/Polymer	PVA-$AgNo_3$	Ag^+	SF 180 i-butene/n-butene	[272]
1.3-butadiene/1-butene	FT/Polymer	AgBF4/Nafion	Ag^+	SF 3.4 1.3-butadiene/1-butene	[112]
c-2-butene/ t-2-butene	FT/Polymer	Nafion membranes	Ag^+	SF 2.7 cis/trans	[273]
di-olefin/mono-olefin	FT/Polymer	Neosepta® CM-1 (Tokuyama)	Ni	SF 70 1.5-hexadiene/1-hexene	[274]
1-hexene/1,5-hexadiene	FT/Polymer	Neosepta® CM-1 (Tokuyama)	Ag^+	NS	[120]
butane/methane	Polymer	PMP/TiO_2	-	SF 34 butane/CH4	[275]
butane isomers	Zeolite	CM7	-	SF > 10	[276]
acetylene/ethylene	FT/Hybrid	RTILs	Cu	SF 23 acetylene/ethylene	[277]
C12 to C33	Adsorbent	Ag^+-SPE	Ag^+	NS	[278]
pentane/octane	FT/Liquid	$[Ag(DMBA)_2][Tf_2N]$	Ag^+	NS	[279]
propane/CH_4	FT/Polymer	PDMS	Ag^+	SF 25 propane/CH4	[280]
1-octane/n-octane	FT/Liquid	bis(trifluoromethylsulfonyl)imide/Ag	Ag^+	S 3.92 1-octane/n-octane	-
propane/ethane	CMS	6FDA/BPDA-DAM	-	S 4.38 propane/ethane	[221]
propylene/ethylene	CMS	6FDA/BPDA-DAM	-	S 0.91 propylene/ethylene	[221]

The separation factor (SF) of the gas pairs may be defined as the quotient between the molar ratios of the components in the permeate side divided by the quotient between the molar ratios of the components in the feed side. The ideal selectivity (S) is calculated as the ratio between the permeances of the individual components. NS stands for not specified.

Table S2. Distribution of membranes and conditions used for paraffin/olefin separations.

Separated Gases	Type of Material	Name of the Material	Carrier	Selectivity or Sep Factor	Permeability or Permeance	Tem p (K)	Pressure (bar)	Flow Rate (mL/min)	Ref.
i-butene/i-butane	FT/Polymer	PTMSP-AgClO4	Ag^+	S 5.6 i-butene/i-butane	P 31.1 i-butene; P 5.6 i-butane [x108 (cm^3 (STP)cm/cm^2.s.cmHg)]	298	1.519	NS	[281]
i-butene/i-butane	Polymer	PTMSP	-	S 3.0 i-butene/i-butane	P 286 i-butene; P 96.5 i-butane [x108 (cm^3 (STP)cm/cm^2.s.cmHg)]	298	1.519	NS	[281]
1,3-butadiene/n-butane	CMS	BPDA-DDBT/DABA	-	SF 50 1,3-butadiene/n-butane	P 80 GPU butane	373	1.013	NS	[82]
1,3-butadiene/n-butane	Polymer	6FDA-TrMPD	-	NS	P 111 ethylene (barrer)	323	2.02	NS	[282]
1-butene/n-butane	FT/Liquid	ILMs in PVDF substrates	Ag^+	SF 850 butene/n-butane	NS	NS	0,14	NS	[256]
1-heptene/n-heptane	FT/Liquid	bis(trifluoromethylsulfonyl)imide/Ag	Ag^+	S 5.13 1-heptene/n-heptane	NS	293	1.013	NS	[283]
1-hexene/n-hexane	FT/Liquid	bis(trifluoromethylsulfonyl)imide/Ag	Ag^+	S 5.15 1-hexene/n-hexane	NS	293	1.013	NS	[283]
1-hexene/n-hexane	FT/Polymer	PDMS/PPSQ membrane	Ag^+	SF 40–70	NS	335	0.005	1.4*10-6 g/s.cm^3	[284]
1-pentene/n-pentane	FT/Liquid	bis(trifluoromethylsulfonyl)imide/Ag	Ag^+	S 9.23 1-pentene/n-pentane	NS	293	1.013	NS	[283]
C_4-C_{10} paraffin/olefin	Adsorbent	zeolite 5A (UOP)	-	98% pure paraffin at the end	NS	593	NS	NS	[210]
c-butene/n-butane	FT/Polymer	PTMSP-AgClO4	Ag^+	SF 1.3 c-butene/n-butane	P 268 c-butene; P 205 n-butane [$\times10^8$ (cm^3 (STP)cm/cm^2.s.cmHg)]	298	1.519	NS	[281]
c-butene/n-butane	Polymer	PTMSP	-	SF 1.2 c-butene/n-butane	P 330 c-butene; P 285 n-butane [$\times10^8$ (cm^3 (STP)cm/cm^2.s.cmHg)]	298	1.519	NS	[281]
cis-2-butene/isobutane	FT/Polymer	PE-g-AA-Ag^+	Ag^+	S 9.2 c-2-butene/i-butane	P 18.6 c-2-butene; P 1.44 i-butane ($\times10^{-10}cm^3$cm/cm^2 cmHg)	303	NS	NS	[285]
cyclohexane/cyclohexene	FT/Hybrid	AgCl//PMMA	Ag^+	NS	NS	NS	NS	NS	[286]
cyclohexene/isooctane	FT/Hybrid	Nafion membranes	Ag^+	S 2.8 styrene	P 3.28 Styrene/ Isooctane (mol cm M^{-1} cm^{-2} s^{-1} × 10^15)	298	1.013	NS	[287]
ethylene/ethane	MOF	6FDA-DAM:DABA	Fe	SF 9 ethylene/ethane	P 90 ethylene (barrer)	308	3.44	NS	[233]
ethylene/ethane	Adsorbent	Ca-ETS-4	-	SF 35 ethylene/ethane	NS	570	NS	NS	[211]
ethylene/ethane	Adsorbent	Ag/SiO2	Ag	NS	NS	348	NS	NS	[288]
ethylene/ethane	CMS	Carbonized BPDA-pp'ODA Polyimide	-	SF 5 ethylene/ethane	P 1 ethylene ($\times10^{-8}$ mol m^{-2} s^{-1})	373	1.013	NS	[81]
ethylene/ethane	CMS	not specified	-	S 5 ethane	NS	259–399	1–10.0	NS	[85]
ethylene/ethane	CMS	Matrimid® 5218 (Huntsman)	-	S 12 ethane	P 14.4 (barrer)	308	NS	NS	[215]
ethylene/ethane	CMS	Matrimid® 5218 (Huntsman)	-	S 12 ethylene/ethane	P 14–15 ethylene (barrer)	308	3.447	NS	[216]

ethylene/ethane	CMS	Matrimid	-	SF 60 ethylene/ ethane	P 4.8 × 10^{-7} ethylene; P 1.6 × 10^{-9} ethane ($mol·Pa^{-1}·m^{-2}·s^{-1}$)	NS	NS	NS	[217]
ethylene/ethane	CMS	6FDA/BPDA-DAM	-	SF >20	P 10 ethylene GPU	308	20.265	NS	[218]
ethylene/ethane	CMS	PIM-6FDA-OH	-	SF 17.5 ethylene/ethane	P 10 ethylene (barrer)	308	20.265	1000 cm^3/min	[219]
ethylene/ethane	CMS	Matrimid and 6FDA/BPDA-DAM	-	NS	NS	308	8.04	NS	[220]
ethylene/ethane	CMS	6FDA/BPDA-DAM	-	S 3.9 ethylene/ethane	P 15.9 ethylene; P 4.0 ethane (GPU)	298	5.15	NS	[221]
ethylene/ethane	FT/Hybrid	not specified	Ag	NS	NS	NS	NS	NS	[125]
ethylene/ethane	FT/Hybrid	not specified	Ag^+	SF 65 ethylene/ethane	P 10^−9 GPU	380	1.013	NS	[126]
ethylene/ethane	FT/Hybrid	Chitosan/Ag (Imtex)	Ag^+	SF 100 ethylene/ethane	P 100 ethylene (barrer)	298	5.15	NS	[127]
ethylene/ethane	FT/Hybrid	5A zeolite	Ag^+	S 27.4 ethylene	NS	298	1	NS	[128]
ethylene/ethane	FT/Hybrid	Fe2(dobdc)	Ag^+	S 13.6 ethylene	NS	298	1	NS	[128]
ethylene/ethane	FT/Liquid	Fluoropore FP-010/AgNO3 (Sumitomo)	Ag^+	SF 460 ethylene/ethane	NS	298	1.01	300 cm^3/min	[90]
ethylene/ethane	FT/Liquid	polysulfone	Ag^+	SF 420 ethylene/ethane	P 5.17 ethylene/ethane (×10^{-3} $cm^3/(cm^2/s)$)	298	6.89	50 cm^3/min	[129]
ethylene/ethane	FT/Liquid	PEO/PBT/ AgNO3	Ag^+	SF 165 ethylene/ethane	P 50 ethylene (barrer)	298	3	100 mL/min	[130]
ethylene/ethane	FT/Liquid	EPDM-SPEEK	Ag^+	SF 2700 ethylene/ethane	P 7.6 ethylene/ethane (× 10^{-10} cm^3/cm^2 s Pa)	298	3	150 mL/min	[131]
ethylene/ethane	FT/Liquid	[4-mebupy]BF4	Ag^+	S 3 ethylene	NS	303	10	NS	[132]
ethylene/ethane	FT/Liquid	Cu SILM supported PVDF	Cu	S 11,8	P 2,653 barrer	NS		60 $mL·min^{-1}$	[133]
ethylene/ethane	FT/Liquid	PIL/40IL–Ag^+ 1.25 M	Ag^+	S 7.24 etylene	P 13.1 GPU etylene/ethane	293	1	NS	[134]
ethylene/ethane	FT/Liquid	ZnCl2/[BMIM][Cl]	Zn IL	S 178	P 25.8 barrer	298	1.1	NS	[135]
ethylene/ethane	FT/Liquid	CuCl/ChCl-EG-based SLMs	Cu	S 12.5	P 122.2 barrer	298	1.1	60 mL/min	[258]
ethylene/ethane	FT/Liquid	CuCl/DESs-SLMs	IL	SF 20 ethylene/ethane	P 13 ethylene; P 1 ethane (barrer)	298	0.1	NS	[135]
ethylene/ethane	FT/Liquid	DESs-SLMs	Ag^+	S 50–100 ethylene	P 800–1200 ethylene/ethane (barrer)	298	0.1	20 mL/min	[111]
ethylene/ethane	FT/Polymer	Nafion N-117	Ag^+	SF 540 ethylene/ethane	P 12,500 (barrer)	298	NS	NS	[137]
ethylene/ethane	FT/Polymer	AgBF4/PVP	Ag^+	SF 2.3 ethylene/ethane	P 0.9 (× 10^{-10} $mol(m^2·s·Pa)^{-1}$)	423	NS	NS	[138]
ethylene/ethane	FT/Polymer	AgBF4/PEO	Ag	SF 240 ethylene/ethane	NS	296	1.72	NS	[139]
ethylene/ethane	FT/Polymer	Pebax® 4011 and Pebax® 2533 (Atofina)	Ag^+	NS	NS	291	NS	NS	[140]
ethylene/ethane	FT/Polymer	AgNO3/polyethersulfone (Daicel)	Ag^+	SF 1100 ethylene/ethane	P 4 (×10^{-5} mol m^{-2} s^{-1} kPa^{-1})	298	0.09	NS	[141]
ethylene/ethane	FT/Polymer	PA 1 2-PTMO/AgBF4	Ag	SF 20 ethylene/ethane	NS	295	3.44	NS	[142]
ethylene/Ethane	FT/Polymer	POZ/AgBF4	Ag^+	SF 5 ethylene/ethane	P 40 ethylene GPU	298	3.77	NS	[143]
ethylene/ethane	FT/Polymer	EPDM	Ag^+	SF 72.5 ethylene/ethene	NS	298	3	NS	[136]
ethylene/ethane	FT/Polymer	AgNO3/polyethersulfone	Ag^+	SF 374 ethylene/ethane	P 3.3 (×10−4 cm^3 cm^{-2} s^{-1} cm Hg^{-1})	298	2	NS	[144]

		(Daicel)							
ethylene/ethane	FT/Polymer	PebaxTM 2533/AgBF4	Ag^+	NS	NS	298	NS	NS	[145]
ethylene/ethane	FT/Polymer	3c	Ag^+	SF 115 ethylene/ethane	P 7.5 ethylene/ethane GPU	298	1.37	NS	[146]
ethylene/ethane	FT/Polymer	SiO2 Poly(sodium acrylate) Ag^+	Ag^+	SF 94 ethylene/ethane	P 30 ($\times 10^{-10}$ mol m^{-2} s^{-1} Pa^{-1})	373	2	NS	[147]
ethylene/ethane	FT/Polymer	Pebax® 2533/AgBF4 (Arkema)	Ag^+	SF 55 ethylene/ethane	P 160 (unit NS)	296	3.44	8 cm^3/min	[115]
ethylene/ethane	FT/Polymer	28% PVDF/72% triacetin/AgNO3	Ag^+	NS	NS	298	NS	NS	[129]
ethylene/ethane	FT/Polymer	Psf/AgNO3	Ag	NS	P 185 ethylene (l/(m^2·h·bar))	NS	1	NS	[148]
ethylene/ethane	FT/Polymer	PSf/PTMSP	Ag	NS	P 83 GPU	NS	NS	NS	[149]
ethylene/ethane	FT/Polymer	AgBF4–PVMK membrane	Ag^+	ethylene/ethane	NS	298	2.06	NS	[150]
ethylene/ethane	FT/Polymer	PEO-AgBF4	Ag	NS	P 0.5–5 propane; 10^{-8} to 10^{-9} propylene [×10−12 cm^3 (STP) cm/(cm^2 s cmHg)]	296	7.9	NS	[151]
ethylene/ethane	Graphene	2N-Pore-13	-	S 1.04 ethylene/ethane	NS	298	NS	NS	[289]
ethylene/ethane	Hybrid	PSf hollow fiber	Ag		P 4.7 GPU	NS	NS	NS	[113]
ethylene/ethane	Hybrid	CA–S30	-	S 2.16 ethylene/ethane	P 0.11 ethane (barrer)	308	2	NS	[213]
ethylene/ethane	MOF	ZIF-7	Zn	NS	NS	NS	0	NS	[77]
ethylene/ethane	MOF	ZIF-8	-	S 2.8 ethylene	P 1.5 ethylene (10^{-8} mol $m^{-2}s^{-1}$ Pa^{-1})	298	1	NS	[78]
ethylene/ethane	MOF	Cu3BTC2	Cu	SF 7.1 ethylene/ethane	P 17 ($\times 10^{-18}$ mol m/(m^2 s Pa)	423	5	NS	[234]
ethylene/ethane	MOF	Cu3BTC2	Cu	SF 7,1 ethylene/ethane	P 17 ($\times 10^{-18}$ mol m/(m^2 s Pa)	423	5	NS	[234]
ethylene/ethane	MOF	IRMOF-8	Zn	S 1.43 Ethane/Ethylene	NS	318	8	NS	[235]
ethylene/ethane	MOF	MIL-101	Cu	SF 16.5 ethylene/ethane	NS	303	1	NS	[236]
ethylene/ethane	MOF	MIL-100	Fe	111 ethylene/ethane	NS	298	0.01	NS	[225]
ethylene/ethane	MOF	M–MOF-74	Co, Fe, Mn, Ni	SF 10 ethylyne/ethane	NS	318	1	NS	[237]
ethylene/ethane	MOF	Mg2(dhtp)	Mg	S 1.4 ethylene/ethane	NS	293	0.015	0.25 mL/min	[238]
ethylene/ethane	MOF	CO2(dhtp)	Co	S 1.7 ethylene/ethane	NS	293	0.015	0.25 mL/min	[238]
ethylene/ethane	MOF	ZIF-8		S 0.48 ethylene/ethane	NS	293	0.015	0.25 mL/min	[238]
ethylene/ethane	MOF	Fe2(dobdc)	Fe	NS	NS	318	NS	NS	[101]
ethylene/ethane	MOF	CuBTC	Cu	NS	NS	303; 373	0.01–5	NS	[239]
ethylene/ethane	MOF	ZIF-71	-	SF 1.84 propane/propylene	NS	293	1	NS	[240]
ethylene/ethane	Polymer	6FDA-1,5-NDA	-	S 5.8 ethylene/ethane	P 2.1 ethylene<br>P 0.5 ethane (unit NS)	308	5.066	NS	[290]
ethylene/ethane	Polymer	PPO homopolymer	-	SF 50 ethylene/ethane	P 15 (× 10^{-10} cm^3 cm/ cm^2 s cmHg)	293	1.82	NS	[291]
ethylene/ethane	Polymer	6FDA	-	SF 3.2 ethylene/ethane	NS	308	NS	NS	[292]
ethylene/ethane	Polymer	6FDA–NDA	-	S 6.84 ethylene/ethane	P 1.17 ethylene<br>P 0.17 ethane (barrer)	308	2.026	NS	[293]

ethylene/ethane	Zeolite	CuCl-modified tubular γ-Al2O3 membrane	Cu$^+$	SF 1.4 ethylene/ethane	NS	333	2.026	NS	[226]
ethylene/ethane	Zeolite	CuCl/NaX	Cu	NS	NS	358	2	8 mL/min	[227]
ethylene/ethane	Zeolite	Na-ETS-10	-	S 5 ethylene	NS	298	1.013	180 mL/min	[228]
ethylene/ethane	Zeolite	AgA and AgX	Ag$^+$	NS	NS	303	1.013	NS	[229]
ethylene/ethane	Zeolite	ZIF-4 and ZIF-zni	-	NS	NS	293	NS	NS	[230]
ethylene/ethane	Zeolite	ZIF-4	-	SF 1.71 ethane/ethylene	NS	293	up to 12	1 mL/min	[231]
ethylene/ethane	Zeolite	Ag-X	Ag	S 15.9 ethylene	P 9.04 [$\times10^{-8}$ mol m^{-2} s^{-1} Pa^{-1}]	303	NS	NS	[232]
hexene/hexane	FT/Liquid	[Ag(olefin)+Tf2N-]	Ag$^+$	SF 163 hexene/hexane	P 123.27 GPU 1-hexene	296	2.12	2.59*10^{-3} mL/scm^2	[294]
i-butane/i-butene	MOF	Cu-BTC MOF	Cu	SF 4.0 i-butane/i-butene	NS	298	1	NS	[295]
i-butene/i-butane	FT/Polymer	(PTMSP-g-AA-Ag$^+$)	Ag$^+$	S 6 i-butene/i-butane	P 10 i-butene; P 1.66 i-butane ($\times10^{-8}$ [cm^3(STP)cm/cm 2 s cmHg]	298	NS	NS	[258]
i-butene/i-butane	FT/Polymer	LLDPE-g-AA-Ag +	Ag$^+$	S 10.3 isobutene	P 3.31 isobutene/isobutane ($\times10^{-10}$ cm^3(STP) cm/cm^2·s)	298	1.013	NS	[296]
i-butene/i-butane	FT/Polymer	SR-g-AA-Ag +	Ag$^+$	SF 2.9 i-butene/i-butane	NS	298	1.013	NS	[297]
i-butene/i-butane	FT/Polymer	PE-g-AA-Ag$^+$	Ag$^+$	S 10.3 i-butane	NS	298	NS	NS	[209]
i-butene/i-butane	FT/Polymer	PE-g-AA-Cu$^+$	Cu$^+$	S 3.4 i-butane	NS	298	NS	NS	[209]
i-butene/i-butane	FT/Polymer	PE-g-AA-Cu2+	Cu2$^+$	S 2.9 i-butane	NS	298	NS	NS	[209]
i-butene/i-butane	MOF	Cu3(BTC)2	Cu	NS	NS	NS	NS	NS	[75]
isoprene/n-pentane	Adsorbent	Cu(I)-olefin nitrate solution	Cu	NS	NS	313	NS	NS	[212]
isoprene/n-pentane	FT/Hybrid	[Bmim]BF4· · ·AgX	Ag$^+$	NS	NS	298	NS	NS	[298]
isoprene/n-pentane	FT/Hybrid	SPEEK-AgNO3 (3M)	Ag$^+$	SF 890 isoprene/n-pentane	NS	333	101.325	80 mL/min	[259]
isoprene/pentane	FT/Polymer	AgBF4-CA	Ag$^+$	SF 83 isoprene/n-pentane	P 3.9 ($\times$ 10^{-5} cm^3 cm^{-2} s^{-1})	298	1	NS	[299]
pentene/pentane	Adsorbent	CuCl	Cu	NS	NS	NS	NS	0.8 cc/min	[72]
pentene/pentane	FT/Liquid	[Ag(olefin)+Tf2N-]	Ag$^+$	SF 546 pentene/pentane	P 137.14 GPU 1-pentene	296	2.12	2.87*10^{-3}	[294]
pentene/pentane	FT/Polymer	FM-127	Ag$^+$	SF 44.0 pentene/pentane	NS	NS	NS	NS	[255]
pentene/pentane	FT/Polymer	Select	Ag$^+$	SF 23 pentene/pentane	P 2.5 pentene ($\times10^{-8}$ cm^2 s^{-1})	298	1.013	NS	[260]
propylene/propane	Absorbent	Ni-MOF-74	Ni	NS	NS	298; 323; 348	NS	8 mL/min	[40]
propylene/propane	Adsorbent	Ag/SiO2	Ag	NS	NS	348	NS	NS	[289]
propylene/propane	Adsorbent	NH-pore-13	-	SF 54 propylene/propane	NS	298	NS	NS	[300]
propylene/propane	CMS	6FDA/BPDA–DDBT copolyimide	-	S 22 propylene	P 26 GPU propylene	373	1.013	NS	[84]
propylene/propane	CMS	NTDA-BAHFDS	-	S 42 propane	P 26 GPU propylene/propane	308	1.013	100mL/min	[86]
propylene/propane	CMS	AlPO-14	-	NS	NS	NS	NS	NS	[222]
propylene/propane	CMS	6FDA/BPDA-DAM	-	S 20.5	P 17.5 propylene; P 0.85 propane	298	5.15	NS	[221]

				propylene/propane	(GPU)				
propylene/propane	CMS	CMS/g-Al2O3	-	SF 36 propylene/propane	P 9 GPU propylene	298	1.3 - 4	50 mL/min	[223]
propylene/propane	CMS	6FDA	-	S 50 - 60 propylene	P 8 propylene/propane [$\times 10^{-9}$ mol/(m^2 s Pa)]	393	6.89	50 mL/min	[224]
propylene/propane	CMS	CMS membranes synthesized on mesoporous g-alumina support	-	SF 31 propylene/propane	P 1.0 [$\times$ 10^{-8} mol s^{-1} Pa^{-1}]	298	3.1	50 mL/min	[63]
propylene/propane	CMS	BPDA-DDBT/DABA	-	SF 13 propylene/propane	P 50 GPU propane	373	1.013	NS	[82]
propylene/propane	FT/Hybrid	Ag/SBA-15	Ag^+	S 10 propylene	NS	323 - 373	vacuum	0.37 - 0.99 cm^3/s (at 298 K)	[152]
propylene/propane	FT/Hybrid	Ag/c-Al2O3	Ag^+	S 1.2 propane	P 2.7 propane/propylene ($\times 10^{-11}$ (molC_3H_8 $s^{-1}m^{-1}Pa^{-1}$)	298 - 323	1.5 - 2	NS	[153]
propylene/propane	FT/Hybrid	POZ/AgNO3/SiO2 (fumed silica nanoparticles) (1:1:0.1)	Ag^+	S 90.0 propylene/propane	P 1.5 GPU	293	2.75	NS	[154]
propylene/propane	FT/Hybrid	POZ/AgNO3/BMIM+NO3-	Ag^+	S 32.0 propylene/propane	P 5.6 (unit NS)	298	NS	NS	[155]
propylene/propane	FT/Hybrid	POZ/AgNO3/BMIM+BF4-	Ag^+	S 31.8 propylene/propane	P 5.4 (unit NS)	298	NS	NS	[155]
propylene/propane	FT/Hybrid	POZ/AgNO3/BMIM+CF3SO3-	Ag^+	S 33.2 propylene/propane	P 5.1 (unit NS)	298	NS	NS	[155]
propylene/propane	FT/Hybrid	PVP/Nano Au (Seahan)	Au	S 22 propylene	P 1.2 GPU propylene/propane	298	1.013	NS	[96]
propylene/propane	FT/Hybrid	POZ	Ag^+	SF 20 -22.5 propylene/propane	P 1.25–1.5 GPU propylene/propane	293	2.75	NS	[156]
propylene/propane	FT/Hybrid	PVDF-HFP/BMImBF4–Ag^+	Ag^+	S 700 propane	P 6,630 propane/propylene (barrer)	293 - 323	0.5 - 3	20 mL/min	[157]
propylene/propane	FT/Hybrid	AgNO3/Al2O3	Ag^+	NS	NS	298	1	20 mL/min	[158]
propylene/propane	FT/Hybrid	MICRODYN MD020 TP 2N	Ag^+	NS	NS	298	1.2	16.66 mL/min	[159]
propylene/propane	FT/Hybrid	TiO2-PEO-AgBF4	Ag^+	S 19 propylene/propane	P 85 propylene (barrer)	298	1	NS	[160]
propylene/propane	FT/Hybrid	Permylene (Imtex)	Ag^+	NS	NS	298	5.56	NS	[161]
propylene/propane	FT/Hybrid	PHMEP-g-PEGBEM/AgBF4/MgO-NS	Ag^+	SF 12.9 propylene/ propane	P 11.8 GPU propylene	298	1.01	NS	[162]
propylene/propane	FT/Liquid	POZ/AgNO3/BMIM+BF4-	Ag^+	SF 31.8 propylene/propane	P 5.6 GPU propylene; P 5.4 GPU propane	NS	NS	NS	[163]
propylene/propane	FT/Liquid	POZ/AgNO3/BMIM+NO3-	Ag^+	SF 32 propylene/propane	NS	273	NS	NS	[163]
propylene/propane	FT/Liquid	zirconia/AgNO3	Ag^+	SF 20 propylene/propane	NS	298	5.15	400 sccm	[164]
propylene/propane	FT/Liquid	TEG/AgBF4	Ag^+	SF 60	P 1 (10^{-7} cm^3 (STP) cm/(cm^2 s	293 -	1.013	66–84	[165]

				propylene/propane	cmHg))	298		cm^3/min	
propylene/propane	FT/Liquid	$AgBF_4$	Ag^+	S 4.5 propylene	P 8 propylene/propane ($\times 10^{-4}$ mol/m^2s)	298	2.2	NS	[166]
propylene/propane	FT/Liquid	PVDF/AgNO3	Ag^+	SF 474 propylene/propane	NS	298	1.2	NS	[167]
propylene/propane	FT/Liquid	BMIM+BF4-/Ag	Ag^+	SF 17 propylene/propane	P 7.8 GPU	NS	2.75	NS	[168]
propylene/propane	FT/Liquid	Ag–BMImBF4	Ag^+	NS	NS	278; 318	NS	NS	[169]
propylene/propane	FT/Liquid	AgNO3/PVDF (Millipore)	Ag^+	NS	NS	298	1.2	NS	[170]
propylene/propane	FT/Liquid	BMIM+BF4	Cu	SF 5.2 propylene/propane	P 4.0 GPU	NS	NS	NS	[93]
propylene/propane	FT/Liquid	PVDF/AgNO3	Ag^+	SF 480 propylene/propane	P 310 propylene (barrer)	298	1.2	NS	[171]
propylene/propane	FT/Liquid	PVDF/AgNO3	Ag^+	SF 490 propylene/propane	P 2.5 GPU propylene	298	1.2	NS	[172]
propylene/propane	FT/Liquid	[Ag(propene)x][Tf2N]	Ag^+	SF 3 propane/propene	P 0. 40 propane (mol cm^{-2} s)	298	1	NS	[173]
propylene/propane	FT/Liquid	RTILs	Ag^+	SF 100 propylene/propane	NS	318	1	NS	[174]
propylene/propane	FT/Liquid	PVDF/AgNO3	Ag^+	SF 270 propylene/propane	P 7 (×10^7 mols/s)	298	1.2	NS	[175]
propylene/propane	FT/Liquid	BMImBF4	Ag^+	SF 20 propylene/propane	P 1.56 propane (E-09 mol/bar m s) without Ag	293	1	NS	[176]
propylene/propane	FT/Liquid	MOIM+NO3-	Ionic liquid	SF 2.8 propylene/propane	P 1.8 (*10^{-6} cm^3(STP)/cm^2 cmHgsec)	308	0.67	NS	[99]
propylene/propane	FT/Liquid	BMIM+BF4-	Ionic liquid	SF 2.3 propylene/propane	P 1.9 (*10^{-6} cm^3(STP)/cm^2 cmHgsec)	308	0.67	NS	[99]
propylene/propane	FT/Liquid	AgNO3 in hollow fiber membrane	Ag^+	75% propylene removal	NS	298	1	10 mL/min	[177]
propylene/propane	FT/Liquid	(Emim,Ag)[BF4]-PICPM+PF6- (Emim,Ag)[Tf2N]- PICPM+Tf2N- (Emim,Ag)[Tf2N]-12HSA	Ag^+	SF 7 propylene/propane	P 70 propylene (barrer)	298	1.013	100 cm^3/min	[178]
propylene/propane	FT/Liquid	MOIM+BF4−/Cu	Cu	SF 2 propylene/propane	P 12 GPU propylene	298	101.325	NS	[179]
propylene/propane	FT/Liquid	PVDF/AgNO3	Ag^+	SF 473.86 propylene/propane	NS	NS	NS	NS	[118]
propylene/propane	FT/Liquid	NMP	Ag^+	S 4.5 propylene	P 8 propylene/propane [$\times 10^{-4}$ mol/(m^2 s Pa)]	293	1.2 - 2.2	0.01 mol/m^2s	[180]
propylene/propane	FT/Polymer	PVA/AgSbF6	Ag^+	S 125 propylene	P 4.1 GPU	298	4.135	NS	[181]
propylene/propane	FT/Polymer	PVDFHFP/BMImBF4/AgBF4	Ag	NS	NS	NS	NS	NS	[182]
propylene/propane	FT/Polymer	PE-g-AA-Ag^+	Cu	SF 21 propylene/propane	P 133 × 10^{-9} propylene; 645 × 10^{-10} propane (unit NS)	313	15.2	NS	[60]
propylene/propane	FT/Polymer	PPO	Ag^+	SF 5.33	P 86.7 propylene/propane (unit	303	2 - 4.0	NS	[183]

				propylene/propane	NS)				
propylene/propane	FT/Polymer	Cu/PVP	Cu	SF 10 propylene/propane	NS	298	1.38	NS	[184]
propylene/propane	FT/Polymer	AgNO3/PEG/Psf	Ag	SF 250 propylene/propane	P 25 GPU	NS	2.76	NS	[185]
propylene/propane	FT/Polymer	AgBF4–PVP	Ag^+	SF 140 propylene/propane	P 7.5 ($\times10^{-8}$ [cm^3(STP)cm/cm^2scmHg]	NS	NS	NS	[124]
propylene/propane	FT/Polymer	POZ	Ag^+	SF 280 proylene/propane	P 100 GPU propylene	296	1.38	8.5 cm^3/min.	[186]
propylene/propane	FT/Polymer	PEO	Ag^+	NS	NS	308	0.0933	NS	[187]
propylene/propane	FT/Polymer	AgBF4-PVP	Ag^+	SF 140 propylene/propane	P 7.5 ($\times10^{-8}$ [cm^3(STP)cm/cm^2 s cmHg]	NS	NS	NS	[188]
propylene/propane	FT/Polymer	AgBF4-POZ	Ag^+	SF 130 propylene/propane	P 15 ($\times10^{-8}$ [cm^3(STP)cm/cm^2 s cmHg]	NS	NS	NS	[188]
propylene/propane	FT/Polymer	PVP/AgBF4	Ag^+	NS	NS	293 - 343	4.14	NS	[189]
propylene/propane	FT/Polymer	PVP/AgBF4	Ag^+	SF 60 propylene/propane	P 32 GPU propylene	NS	2.76	NS	[190]
propylene/propane	FT/Polymer	PVP/AgNO3/Ppy	Ag^+	NS	NS	NS	2.76	NS	[191]
propylene/propane	FT/Polymer	POZ	Ag^+	SF 5 propylene/propane	P 30 GPU propylene	298	4.136	NS	[192]
propylene/propane	FT/Polymer	PEP/AgBF4	Ag^+	SF 55 propylene/propane	P 6.5 GPU propylene	293	2.758	NS	[94]
propylene/propane	FT/Polymer	PDMS/AgBF4	Ag^+	SF 200 propylene/propane	P 15 GPU propylene	NS	1.38	NS	[193]
propylene/propane	FT/Polymer	PHMV	Ag^+	S 336 propylene	P 33.6 propylene/propane (barrer)	296	1.013	NS	[194]
propylene/propane	FT/Polymer	POZ	Ag^+	SF 65 propylene/propane	P 18 GPU	298	NS	NS	[195]
propylene/propane	FT/Polymer	PVP/silver salts	Ag^+	NS	NS	298	2.76	NS	[196]
propylene/propane	FT/Polymer	POZ/AgBF4	Ag^+	SF 45 propylene/propane	P 10 GPU propylene	298	3.77	NS	[197]
propylene/propane	FT/Polymer	6FDA–4MPD/DABA	Ag	S 10 propylene/propane	P 405 GPU propylene	308	6	NS	[198]
propylene/propane	FT/Polymer	BMIM+BF4	Ag^+	SF 17 propylene/propane	P 8 GPU propylene	NS	NS	NS	[95]
propylene/propane	FT/Polymer	SBS/0.5Ag	Ag^+	S 80 propylene/propane	P 5 GPU	NS	NS	NS	[199]
propylene/propane	FT/Polymer	Ag–sugar/BMIM+BF4 − (0.05/1)	Ag^+	SF 12.9 propylene/propane	P 3.1 GPU	298	NS	NS	[200]
propylene/propane	FT/Polymer	PVC-g-P4VP	Ag^+	S 6 propylene	P 5.7 GPU propylene/propane	298	2.75	NS	[201]
propylene/propane	FT/Polymer	PEI/Pebax2533/AgBF4	Ag^+	SF 1000 propylene/propane	P 40 GPU propylene	298	2–8.0	NS	[202]
propylene/propane	FT/Polymer	PU/AgCF3SO3 (BASF)	Ag^+	S 10 propylene	P 188 propylene/propane (barrier)	298	2	NS	[203]
propylene/propane	FT/Polymer	PTFE (Mencor)	Ag^+	60% propylene	NS	298	1.2	400 mL/min	[121]
propylene/propane	FT/Polymer	PP/AgBF4	Ag^+	NS	NS	298	1.2	16	[204]

								mL/min	
propylene/propane	FT/Polymer	polymer membranes with inorganic nanoparticles uniformly dispersed	Zn	SF 18.08 propylene/propane	P 20.5 propylene (barrer)	NS	NS	NS	[205]
propylene/propane	FT/Polymer	Pebax® 1657/AgBF4 (Atofina)	Ag^{+}	SF 20.4 propylene/propane	P 123.3 peopylene (barrer)	303	2	NS	[206]
propylene/propane	FT/Polymer	poly(vinylalcohol)/AgBF4/Al(NO3)3	Ag^{+}	SF 17 propylene/propane	P 11 GPU propylene	NS	3	NS	[98]
propylene/propane	FT/Polymer	(PVA)/AgBF4/Al(NO3)3	Ag	NS	P 11 GPU	NS	3	NS	[98]
propylene/propane	FT/Polymer	PVP/AgBF4/Al(NO3)3/Ag2O	Ag^{+}	SF 21.7 propylene/propane	P 4.3 GPU	NS	NS	NS	[100]
propylene/propane	FT/Polymer	CAF (CMS)	Ag	SF 50 propylene/propane	P 200 GPU propylene	298	5.15	NS	[207]
propylene/propane	FT/Polymer	SBS/Cu@MIL-101(Cr) MMM	Cu	S 2 propylene	NS	308	NS	NS	[208]
propylene/propane	FT/Polymer	PE-g-AA-Ag^{+}	Ag^{+}	S 5 propane	NS	298	NS	NS	[209]
propylene/propane	FT/Polymer	PE-g-AA-Cu^{+}	Cu^{+}	S 2.2 propane	NS	298	NS	NS	[209]
propylene/propane	FT/Polymer	PE-g-AA-Cu2+	$Cu2^{+}$	S 1.7 propane	NS	298	NS	NS	[209]
propylene/propane	FT/Polymer	PEO-AgBF4	Ag	NS	P 0.5–5 propane; 10^{-8} to 10^{-9} propylene [× 10^{-12} cm^3 (STP) cm/(cm^2 s cmHg)]	296	7.9	NS	[151]
propylene/propane	Hybrid	BTESM	-	SF 8.8 propylene/propane	P 6.32 propylene (x10^{-7} $molm^{-2}$ s^{-1} Pa^{-1})	323	2	NS	[214]
propylene/propane	Hybrid	BTESM	-	SF 30 propylene/propane	P 0.495 propylene [10^{-7} mol m^{-2} s^{-1} Pa^{-1}]	323	2	NS	[301]
propylene/propane	Hybrid	cellulose acetate	-	NS	NS	344,3	NS	2,764 kmol/h	[302]
propylene/propane	Hybrid	PES–PEBA–ZIF-8–APTES		S 3.26 propylene	NS	NS	4	NS	[303]
propylene/propane	MOF	Mg2(dhtp)	Mg	S 1.7 propylene/propane	NS	293	0.015	0.25 mL/min	[238]
propylene/propane	MOF	CO2(dhtp)	Co	S 2.9 propylene/propane	NS	293	0.015	0.25 mL/min	[238]
propylene/propane	MOF	ZIF-8		S 0.7 propylene/propane	NS	293	0.015	0.25 mL/min	[238]
propylene/propane	MOF	Fe2(dobdc)	Fe	NS	NS	318	NS	NS	[101]
propylene/propane	MOF	CuBTC	Cu	NS	NS	303; 373	0.01-5	NS	[239]
propylene/propane	MOF	ZIF-8	-	NS	NS	NS	1	50 ml min−1	[241]
propylene/propane	MOF	Basolite® C300 (BASF)	Cu	NS	NS	323 - 373	5	NS	[79]
propylene/propane	MOF	6FDA-Durene/DABAco-polyimides ZIF-8	-	SF 27.38 propylene/propane	NS	308	10.13	NS	[242]
propylene/propane	MOF	NbOFFIVE-1-Ni (also referred	-	NS	NS	298	1	4 cm^3/min	[243]

		to as KAUST-7)							
propylene/propane	MOF	MgPIM	Mg	S 15.23 propylene/propane	P 568 propylene (barrer)	308	9	NS	[304]
propylene/propane	MOF	ZIF-9	-	SF 1.39 ethane/ethylene	NS	293	1	NS	[240]
propylene/propane	MOF	Zr-fum-fcu-MOF	-	NS	NS	328	NS	NS	[80]
propylene/propane	not specified	not specified	-	SF 16.15 propylene/propane	P 38.83 GPU	344.3	0.031	2764.29 kmol/h	[302]
propylene/propane	Polymer	6FDA-1,5-NDA	-	S 10.0 propylene/propane	P 0.89 propylene P 0.05 propane (unit NS)	308	5.066	NS	[290]
propylene/propane	Polymer	PEO	-	S 3.1 propylene/propane	P 45 propylene; P 17 propane (barrer)	308	NS	NS	[103]
propylene/propane	Polymer	6FDA–NDA	-	S 10.1 propylene/propane	P 0.32 propylene P 0.03 propane (barrer)	308	2.026	NS	[293]
propylene/propane	Polymer	Polyimide membrane (UBE)	-	SF 7.0 propylene/propane	NS	NS	4.05	NS	[305]
propylene/propane	Polymer	6FDA-TrMPD	-	SF 1.2 propylene/propane	P 30 propylene (barrer)	323	2.02	NS	[306]
propylene/propane	Polymer	EC	-	SF 3.25 propylene/propane	P 52 propylene (x10^{-10} (cc cm)/(cm^2 s cmHg))	303	3.92	10-15 ml/min	[307]
propylene/propane	Polymer	Matrimid® 5218	-	S 16 propane	P 0.1 GPU propane/propylene	299	1	170 mL/min	[308]
propylene/propane	Polymer	6FDA/BPDA–DDBT copolyimide	-	SF 15 propylene/propane	P 3.6 GPU propylene	373	1	NS	[309]
propylene/propane	Polymer	(CH3)(C3H7)Si–O–	-	S 9.1 propane	P 29.6 GPU C3H6/C3H8	308	NS	NS	[310]
propylene/propane	Polymer	6FDA-DDBT	-	S 20 propylene/propane	P 1.8 propylene (barrier)	323	2.026	NS	[53]
propylene/propane	Polymer	PAEK/Azide (80:20)	-	SF 44 propylene/propane	P 48 propylene (barrer)	308	3.546	NS	[311]
propylene/propane	Polymer	6FDA–6FpDA polyimide	-	SF 20 propylene/ propane	NS	308	1.03	NS	[312]
propylene/propane	Polymer	6FDA-DAM polyimide/ZIF-8	-	S 122 propylene/propane	P 277 propylene (barrer)	308	20.265	NS	[313]
propylene/propane	Polymer	6FDA-Durene/DABA co-polyimides grafted with a, and b-cyclodextrin	-	SF 18.09 propylene/propane	P 521 propylene; P 28 propane (unit NS)	308	10.13	NS	[314]
propylene/propane	Polymer	Celgard X30-240 (Celgard)	-	NS	NS	343	9.322	250 g/min	[315]
propylene/propane	Polymer	PIM-6FDA-OH	-	SF 15 propylene/propane	P 2 propylene (barrer)	308	2	NS	[316]
propylene/propane	Polymer	6FDA-6FpDA	-	S 13 propylene	P 5.5 GPU propylene/ propane	308 - 343	3.102	NS	[317]
propylene/propane	Polymer	Ethyl cellulose (EC) incorporated in nanoporous graphene	-	SF 11.09 propylene/propane	P 2.2 GPU propylene	NS	NS	NS	[318]
propylene/propane	Polymer	P84/Matrimid	-	S 18 propylene/propane at 1.6 bar and 38h	P 0.015 propane; P 0.4 GPU propylene at 4.0 bar (GPU)	308	1.6; 2.5; 4; 5	NS	[319]

propylene/propane	Zeolite	R-Al2O3	-	S 14 propylene/propane at 2.5 bar and 38h S 12 propylene/propane at 4.0 bar and 30h S 13.7 propylene/propane	P 300 ethylene (barrer)	373	1.01	80 mL min-1	[320]
propylene/propane	Zeolite	SiCHA and DD3R	-	SF 104 propylene/propane	NS	323	NS	NS	[321]
propylene/propane	Zeolite	DD3R	-	NS	NS	318	1.2	8 mL/min	[322]
propylene/propane	Zeolite	DD3R	-	NS	NS	383	1.2	8 mL/min	[322]
propylene/propane	Zeolite	ZIF-8	-	NS	NS	303	0.799	NS	[104]
propylene/propane	Zeolite	ZIF-8	-	SF 45 propylene/propane	P 206 propylene (barrer)	298	NS	NS	[323]
propylene/propane	Zeolite	ZIF-8	-	SF 80 propylene/propane	P 120 propylene (barrer)	298	1.013	100 c3/ min	[324]
Propylene/propane	Zeolite	ZIF-8	-	S 160 propylene	P 50 GPU propylene/propane	298	1	NS	[325]
propylene/propane	Zeolite	ZIF-8/PVAc MMMs	-	NS	NS	308	NS	NS	[326]
propylene/propane	Zeolite	AgZM	Ag	S 2.4-4.8 propylene	P 1200 (+/−500) GPU	323 - 348	NS	60 ml/min	[327]
propylene/propane	zeolite	DD3R and SAPO-34		NS	NS	NS	NS	NS	[328]
propylene/propane	Zeolite	Ag-X	Ag	S 55.4 propylene	P 4.13 [$\times 10^{-8}$ mol m^{-2} s^{-1} Pa^{-1}]	353	NS	NS	[231]
propylene/propane	Zeolite	ZIF-4	-	SF 1.06 propane/propylene	NS	293	up to 8	1 mL/min	[231]
t-2-butene/isobutane	FT/Polymer	PE-g-AA-Ag^+	Ag^+	S 12.5 (t-2- butene/i-butane)	P t-butene 13.3 i-butane 1.44 ($\times 10^{-10}$$cm^3$cm/$cm^2$ cmHg)	303	NS	NS	[285]
t-2-butene/n-butane	FT/Polymer	SR-g-AA-Ag^+	Ag^+	SF 4.0 t-2-butene/n-butane	NS	298	1.013	NS	[297]
t-butene/n-butane	FT/Polymer	PTMSP-AgClO4	Ag^+	SF 2.0 t-butene/n-butane	P 405 t-butene P 205 n-butane [$\times 10^8$ (cm^3 (STP)cm/cm^2.s.cmHg)]	298	1.519	NS	[281]
t-butene/n-butane	Polymer	PTMSP	-	SF 1.7 t-butene/n-butane	P 489 t-butene P 285 n-butane [$\times 10^8$ (cm^3 (STP)cm/cm^2.s.cmHg)]	298	1.519	NS	[281]

The separation factor (SF) of the gas pairs may be defined as the quotient between the molar ratios of the components in the permeate side divided by the quotient between the molar ratios of the components in the feed side. The ideal selectivity (S) is calculated as the ratio between the permeances of the individual components. NS stands for not specified.

Table S3. Papers analyzed in the bibliometric study.

Author	Country	Article	Year
Stern, E.W.	USA	Olefin-Paraffin Separation by Suported Cuprous Chloride	1962
Ho,W.S.W., Doyle,G.,Savage,D.S.,Pruettt,R.L.	USA	Olefin Separations via Complexation with Cuprous Diketonate	1988
Teramoto, M. Matsuyama, H., Yamashiro, T., Okamoto, S.	Japan	Separation of Ethylene from Ethane by a Flowing Liquid Membrane Using Silver Nitrate as a Carrier	1989
Lee, K.R., Hwang, S.	USA	Separation of propylene and propane by polyimide hollow-fiber membrane module	1992
Eriksen, O.I., Aksnes, E.,Dahl, I.M.	Norway	Facilitated transport of ethene through Nafion membranes. Part II. Glycerine treated, water swollen membrane	1993
Hsiue, G.; Yang, J.S.	Taiwan	Novel methods in separation of olefin/paraffin mixtures by functional polymeric membranes	1993
Eldridge, R.B.	USA	Olefin/Paraffin Separation Technology: A Review	1993
Ilinich, O.M., Zamaraev, K.I.	Russia	Separation of ethylene and ethane over polyphenyleneoxides membranes: transient increase of selectivity	1993
Funke, H.H., Noble, R.D., Koval, C.A.	USA	Separation of gaseous olefin isomers using facilitated transport membranes	1993
Hsiue, G. and Yang, J.	Taiwan	Ag^{+} Contained Complex Membrane for the Separation of C4 olefin/Paraffin Mixture	1994
HO, W.S., Dalrymple, D.C.	USA	Facilitated transport of olefins in Ag^{+}-containing polymer membranes	1994
TSOU, D.T., Blachman,M.W., David,J.C.	USA	Silver-Facilitated Olefin/paraffin Contactor System Separation in a Liquid Membrane	1994
Verma, V.K., Phillips, C., Dinh, C.	USA	Revamping Olefins Plant with Membrane Technology	1994
Hayashi, J., Mizuta, H., Yamamoto, M., Kusakabe, K., Morooka, S.	Japan	Separation of Ethane/Ethylene and Propane/Propylene Systems with a Carbonized BPDA-pp'ODA Polyimide Membrane	1996
Yang, J., Hsiue, G.	Taiwan	C4 olefin/paraffin separation by poly[(1-trimethylsilyl)-1-propyne]-graft-poly(acrylic acid)-Ag + complex membranes	1996
Barchas, R., Wallsgrove, C.	USA	Facilitated Transport Membranes for Olefin Separation	1996
Yang, J., Hsiue, G.	Taiwan	Novel dry poly[(1-trimethylsilyl)-1-propyne]-AgC10 4 complex membranes for olefin/paraffin separations	1996
Yamaguchi, T., Baertsch, C., Koval, C.A., Noble, R.D., Bowman, C.N.	USA	Olefin separation using silver impregnated ion-exchange membranes and silver salt/polymer blend membranes	1996
Tanaka,K., Taguchi,A., Hao, H. J., Okamoto,K.K.	Japan	Permeation and separation properties of polyimide membranes to olefins and paraffins	1996
Hsiue, G.; Yang, J.S.	Taiwan	Polymeric Complex Membranes for Olefin/Paraffin Separation	1996
Yang, J., Hsiue, G.	Taiwan	Selective olefin permeation through Ag(I) contained silicone rubber-graft-poly(acrylic acid) membranes	1996
Al-Rabiah, A., Timmerhaus, K., Noble, R.	USA	Utilization of a Hybrid Membrane System in Olefin Production	1996
van Zyl, A. J., Kerres, J. A., Cui, W., Junginger, M.	South Africa	Application of new sulfonated ionomer membranes in the separation of pentene and pentane by facilitated transport	1997
Zy,A.J., Linkov,V.M.	South Africa	Influence of oxygen-containing hydrocarbons on the separation of olefin/paraffin mixtures using facilitated transport	1997
Okamoto,K., Noborio,K., Hao,J., Tanaka,K., Kita,H.	Japan	Permeation and separation properties of polyimide membranes to 1,3-butadiene and n-butane	1997
Bai, S., Sridhar,S., Khan, A.,A.	India	Metal-ion mediated separation of propylene from propane using PPO membranes	1998
Bessarabov, D. G., Theron, J. P., Sanderson, R. D.	South Africa	Novel application of membrane contactors: solubility measurements of 1-hexene in solvents containing silver ions for liquid olefin/paraffin separations	1998
Goering, R. M., Bowman, C.N., Koval, C.A., Noble, R.D., Williamson, D.L.	USA	Role of ion-exchange membrane morphology and sorption properties in facilitated transport di-olefin/mono-olefin separations	1998
Yang,J., Hsiue,G.	Taiwan	Swollen polymeric complex membranes for olefin/paraffin separation	1998

Rege, S.U., Padin, J., Yang, R.T.	USA	Olefin/ Paraffin Separations by Adsorption: complexation vs. Kinetic Separation	1998
Safarik, D.J., Eldridge, R.B.	USA	Olefin/Paraffin Separations by Reactive Absorption: A Review	1998
Okamoto, K., Kawamura, S., Yoshino, M., Kita, H., Hirayama, Y., Tanihara, N., Kusuki, Y.	Japan	Olefin/Paraffin Separation through Carbonized Membranes Derived from an Asymmetric Polyimide Hollow Fiber Membrane	1999
Lin, Y. S., Ji, W., Wang, Y., Higgins, R. J.	USA	Cuprous-Chloride-Modified Nanoporous Alumina Membranes for Ethylene-Ethane Separation	1999
Bessarabov, D. G., Theron, J.P., Sanderson, R.D., Schwarz, H.-H., Schossig-Tiedemann, M., Paul, D.	South Africa	Separation of 1-hexene/n-hexane mixtures using a hybrid membrane/extraction system	1999
Sridhar, S., Khan, A.A.	India	Simulation studies for the separation of propylene and propane by ethylcellulose membrane	1999
Kim, Y.H., Ryu, J.H., Bae, J.Y., Kangb, Y.S., Kim, H.S.	Korea	Reactive polymer membranes containing cuprous complexes in olefin/paraffin separation	1999
Goering, R. M., Bowman, C. N., Koval, C. A., Noble, R. D., Ashley, M. E.	USA	Complexation structure and transport mechanism of 1,5-hexadiene and 1-hexene through silver facilitated transport membranes	2000
Staudt-Bickel, C., Koros, W.J.	Germany	Olefin/paraffin gas separations with 6FDA-based polyimide membranes	2000
Krol, J.J., Boerrigter,M., Koops,G.H.	Netherlands	Polyimide hollow fiber gas separation membranes: preparation and the suppression of plasticization in propane/propylene environments	2000
Kim, H. S., Ryu, J. H., Kim, H., Ahna, B. S., Kang, Y. S.	South Korea	Reversible olefin complexation by silver ions in dry poly(vinyl methyl ketone) membrane and its application to olefin/paraffin separations	2000
Park, Y.S., Won, J., Kang, Y.S.	Korea	Preparation of Poly(ethylene glycol) Brushes on Polysulfone Membranes for Olefin/Paraffin Separation	2000
Koros, W.J., Mahajan, R.	USA	Pushing the limits on possibilities for large scale gas separation: which strategies?	2000
Menendez, I., Fuertes, A. B.	Spain	Aging of carbon membranes under different environments	2001
Jose, B., Ryu, J.H., Lee, B.G., Lee, H., Kangb, Y.S., Kim, H.S.	South Korea	Effect of phthalates on the stability and performance of AgBF4–PVP membranes for olefin/paraffin separation	2001
Hong, S.U, Kim, J. Y., Kang, Y.S.	South Korea	Effect of water on the facilitated transport of olefins through solid polymer electrolyte membranes	2001
Su, Caili, Kuraoka, K., Yazawa, T.	Japan	Ethene/Ethane (C2H4/C2H6) Separation through an Inorganic–Organic Hybrid Membrane Containing Silver(I) Ions as Olefin Carriers, Using Poly(N-vinylpyrrolidone) as a mediation Agent	2001
Da Costa, A., Wijmans, H., Baker, R.W.	USA	Ethylene Recovery by membrane technology	2001
Sunderrajan, S., Freeman, B.D., Hall, C.K., Pinnau,I.	USA	Propane and propylene sorption in solid polymer electrolytes based on poly(ethylene oxide) and silver salts	2001
Sungpet, A., Way, J.D., Koval, C.A., Eberhart, M.E.	Thailand	Silver doped Nafion-poly(pyrrole) membranes for facilitated permeation of liquid-phase olefins	2001
Pinnau,I. Toy, L.	USA	Solid polymer electrolyte composite membranes for olefin/paraffin separation	2001
Chan, S. S., Wang, R., Chunga, T., Liu, Y.	Singapore	C2 and C3 hydrocarbon separations in poly(1,5-naphthalene-2,2 -bis(3,4-phthalic) hexafluoropropane) diimide (6FDA-1,5-NDA) dense membranes	2002
Chang, J., Marrero, T.R., Yasudea, H.K.	USA	Continuous process for propylene/propane separation by use of silver nitrate carrier and zirconia porous membrane	2002
Kim, J. H., Min, B. R., Lee, K. B., Wonc, J., Kang Y. S.	South Korea	Coordination structure of various ligands in crosslinked PVA to silver ions for facilitated olefin transport	2002
Müller, J; Peinemann, K.V, Müller, J.	Germany	Development of facilitated transport membranes for the separation of olefins from gas streams	2002
Jose, B., Ryu, J.H., Kim, Y.J., Lee, H.S.D., Kim, H.S.	South Korea	Effect of Plasticizers on the Formation of Silver Nanoparticles in Polymer Electrolyte Membranes for Olefin/Paraffin Separation	2002
Teramoto, M., Takeuchi, N., Maki, T., Matsuyama, H.	Japan	Ethylene/ethane separation by facilitated transport membrane accompanied by permeation of aqueous silver nitrate solution	2002
Kovvali, A.S., Chen, H., Sirkar, K.K.	USA	Glycerol-based Immobilized Liquid Membranes for Olefin-Paraffin Separation	2002

Morisatoa, A., Hea, Z., Pinnau, I., Merkelb, T.C.	USA	Transport properties of PA 12-PTMO/AgBFd solid polymer electrolyte membranes for olefin/paraffin separation	2002
Kim, J. H., Min, B. R., Won, J., Kang, Y. S.	South Korea	Anomalous temperature dependence of facilitated propylene transport in silver polymer electrolyte membranes	2003
Burns, R.L, Koros, W.J.	USA	Defining the challenges for C3H6/C3H8 separation using polymeric membranes	2003
Park, H.H., Won, J., Ohc, S., Kang, Y.	South Korea	Effect of nonionic n-octyl-d-glucopyranoside surfactant on the stability improvement of silver polymer electrolyte membranes for olefin/paraffin separation	2003
Kim,J.H., Min,B,R., Kim,H.S., Won,J., Kang,Y.S.,	South Korea	Facilitated transport of ethylene across polymer membranes containing silver salt: effect of HBF4 on the photoreduction of silver ions	2003
Yoshino, M., Nakamura, S., Kita, H., Okamotoa, K., Tanihara, N., Kusuki, Y.	Japan	Olefin/paraffin separation performance of carbonized membranes derived from an asymmetric hollow fiber membrane of 6FDA/BPDA–DDBT copolyimide	2003
Chan, S. S., Chung, T., Liu, Y., Wang, R.	Singapore	Gas and hydrocarbon (C2 and C3) transport properties of co-polyimides synthesized from 6FDA and 1,5-NDA (naphthalene)/Durene diamines	2003
Yoshino, M., Nakamuraa S., Kita, H., Okamotoa, K., Tanihara, N., Kusuki, Y.	Japan	Olefin/paraffin separation performance of asymmetric hollow fiber membrane of 6FDA/BPDA–DDBT copolyimide	2003
Semenova,S.I.	Russia	Polymer membranes for hydrocarbon separation and removal	2003
Kim, J. H., Min, B. R., Won, J., Kang, Y. S.	South Korea	Revelation of Facilitated Olefin Transport through Silver-Polymer Complex Membranes Using Anion Complexation	2003
Duan, S., Ito, A., Ohkawa, A.	Japan	Separation of propylene/propane mixture by a supported liquid membrane containing triethylene glycol and a silver salt	2003
Al-Rabiah, A.A.	Saudi Arabia	The Use of Hybrid Membrane/Distillation System for the Ethane/Ethylene Separation in Olefin Plants	2003
Nymeijer, D.C., Visser, T., Assen, R., Wessling, M.	Netherlands	Composite hollow fiber gas–liquid membrane contactors for olefin/paraffin separation	2004
Kim, J.H., Park, S.M., Won, J., Kang, Y.S.	South Korea	Dependence of facilitated olefin transport on the thickness of silver polymer electrolyte membranes	2004
Lin,H., Freeman, B.D.	USA	Gas solubility, diffusivity and permeability in poly(ethylene oxide)	2004
Kang, S.W., Kim, J.H., Oh, K.S., Won, J., Char,K., Kim, H.S., Kang,Y.S.	South Korea	Highly stabilized silver polymer electrolytes and their application to facilitated olefin transport membranes	2004
Kim,S.H., Kim, D.B., Choi, D.K., Lee, H., Kim, H.S., Won, J.	South Korea	Isoprene/pentane separation using facilitated transport membranes	2004
Grande, C. A., Araujo, J. D. P., Cavenati, S., Firpo, N., Basaldella, E., Rodrigues, A. E.	Portugal	New π-Complexation Adsorbents for Propane-Propylene Separation	2004
Kima, J.H., Won, J. Kanga, Y.S.	South Korea	Olefin-induced dissolution of silver salts physically dispersed in inert polymers and their application to olefin/paraffin separation	2004
Nymeijer, K., Visser, T., Assen, R. Wessling, M.	Netherlands	Olefin-Selective Membranes in Gas-Liquid Membrane Contactors for Olefin/Paraffin Separation	2004
Kima, J.H., Won,J.,Kang,J.S.	South Korea	Silver polymer electrolytes by _x005F_x0001_-complexation of silver ions with polymer containing C=C bond and their application to facilitated olefin transport membranes	2004
Nymeijer, K., Visser, T., Assen, R., Wessling, M.	Netherlands	Super selective membranes in gas–liquid membrane contactors for olefin/paraffin separation	2004
Liu, L., Feng, X., Chakma, A.	Canada	Unusual behavior of poly(ethylene oxide)/AgBF4 polymer electrolyte membranes for olefin–paraffin separation	2004
Won, J., Kim D. B., Kang, Y. S., Choi, D. K., Kim, H. S., Kim, C. K., Kim, C. K.	South Korea	An ab initio study of ionic liquid silver complexes as carriers in facilitated olefin transport membranes	2005
Kang. S.W., Kim.J.H., Won.J., Char.K., Kang.Y.S.	South Korea	Effect of amino acids in polymer/silver salt complex membranes on facilitated olefin transport	2005
Teramoto, M., Shimizua, S., Matsuyama, H., Matsumiya, N.	Japan	Ethylene/ethane separation and concentration by hollow fiber facilitated transport membrane module with permeation of silver nitrate solution	2005
Curbelo, S., Müller, E. A.	Venezuela	Modelling of Ethane/Ethylene Separation Using Microporous Carbon	2005

Islam, M.N., Zhou, W., Honda,T.,Tanaka, K., Kita, H., Okamoto,K.	Japan	Preparation and gas separation performance of flexible pyrolytic membranes by low-temperature pyrolysis of sulfonated polyimides	2005
Stoitsas, K.A., Gotzias,A., Kikkinides, E.S., Steriotis, T.A., Kanellopoulos, N.K., Stoukides, M., Zaspalis, V.T.	Greece	Porous ceramic membranes for propane–propylene separation via the p-complexation mechanism: unsupported systems	2005
Kim, J. H., Kim, C. K., Won, J., Kang, Y. S.	South Korea	Role of anions for the reduction behavior of silver ions in polymer/silver salt complex membranes	2005
Son, S. J., Choi, H. W., Choi, D. K., Lee, S. D., Kim, H. S., Kim, S. W.	South Korea	Selective Absorption of Isoprene from C5 Mixtures by π Complexation with Cu(I)	2005
Giannakopoulos, I.G., Nikolakis,V.	Greece	Separation of Propylene/Propane Mixtures Using Faujasite-Type Zeolite Membranes	2005
Kim,J.H., Park,S.M., Won,J., Kang,Y.S.	South Korea	Unusual separation property of propylene/propane mixtures through polymer/silver complex membranes containing mixed salts	2005
Van Miltenburg, A., Zhu, W., Kapteijn, F., Moulijn, J.A.	Netherlands	Adsorptive separation of light olefin/paraffin mixtures	2006
Kang, S. W., Char, K., Kim, J. H., Kim, C. K., Kang, Y. S.	South Korea	Control of Ionic Interactions in Silver Salt-Polymer Complexes with Ionic Liquids: Implications for Facilitated Olefin Transport	2006
Hamouda, S. B., Nguyen, Q. T., Langevin, D., Schaetzel, P., Roudesli, S.	France	Fine characterization of the ethylene and ethane sorption in poly(amide 12-block-tetramethylenoxide) copolymer/AgBF4 membranes	2006
Choi, H.W., Kim, D.B., Choi, D.K., Ahm,B.S., Kim, H.G., Lee, C.H., Sung, J.Y.	South Korea	Highly selective facilitated transport membranes for isoprene/n-pentane separation	2006
Yang,D., Barbero,R.S, Devlin,D.J., Cussler,L.,Colling,C.W.,Carrera, M.E.	USA	Hollow fibers as structured packing for olefin/paraffin separations	2006
Kang, S. W., Kim, J. H., Char, K., Won, J., Kang, Y. S.	South Korea	Nanocomposite silver polymer electrolytes as facilitated olefin transport membranes	2006
Arruebo, M., Falconer, J. L., Noble, R. D.	USA	Separation of binary C5 and C6 hydrocarbon mixtures through MFI zeolite membranes	2006
Hess,S., Staudt-Bickel, C.,Lichtenthaler, R.N.	Germany	Propene/propane separation with copolyimide membranes containing silver ions	2006
Yave, W., Shishatskiy, S., Abetz, V., Matson, S., Litvinova, E., Khotimskiy, V., Peinemann, K.	Germany	A Novel Poly(4-methyl-2-pentyne)/TiO2 Hybrid Nanocomposite Membrane for Natural Gas Conditioning: Butane/Methane Separation	2007
Sklari, S.D., Zaspalis, V.T.	Greece	A novel system of Al100P60Oz microporous ceramic membrane for hydrogen separation from hydrogen/propane mixtures	2007
Ruthven, D. M., Reyes, S. C.	USA	Adsorptive separation of light olefins from paraffins	2007
Yang,D., Devlin,D.J., Barbero, R.S.	USA	Effect of hollow fiber morphology and compatibility on propane/propylene separation	2007
Chung, T., Jiang, L.Y., Li, Y., Kulprathipanja,S.	Singapore	Mixed matrix membranes (MMMs) comprising organic polymers with dispersed inorganic fillers for gas separation	2007
Chilukuri, P., Rademakers,K., Nymeijer, K., Ham, L.V., Berg,L.V.	Netherlands	Propylene/Propane Separation with a Gas/Liquid Membrane Contactor Using a Silver Salt Solution	2007
Atchariyawut,S., Jiraratananon,R, Wang,R.	Thailand	Separation of CO2 from CH4 by using gas liquid membrane contacting process	2007
Kim, H.S., Bae, J.Y., Park, S.J., Lee, H., Bae, H.W., Kang, S.O., Lee, S.D., Choi, D.K.	South Korea	Separation of Olefin/Paraffin Mixtures Using Zwitterionic Silver Complexes as Transport Carriers	2007
Merkel, T., Blanc, R., Zeid, J., Suwarlim, A., Firat, B., Wijmans, H., Asaro, M., Greene, M.	USA	Separation of Olefin/Paraffin Mixtures with Carrier Facilitated Membranes	2007
Tomita, T., Suzuki, K., Nakayama, K., Yajima, K.,	Japan	Synthesis and Permeation Properties of a DDR-Type Zeolite Membrane for Separation of CO2/CH4 Gaseous	2007

Yoshida, S.		Mixtures	
Shen,J., Zheng,X., Ruan,H., Wu,L., Qiu,J., Gao,C.	China	Synthesis of AgCl/PMMA hybrid membranes and their sorption performance of cyclohexane/cyclohexene	2007
Hrabáneka, P., Zikánováa, A., Bernauerb, B., Fílab, V., Kočiříka, M.	Czech Republic	A route to MFI zeolite-α-alumina composite membranes for separation of light paraffins	2008
Gascon, J., Blom, W., Miltenburg, A., Ferreira, A., Berger, R., Kapteijn, F.	Netherlands	Accelerated synthesis of all-silica DD3R and its performance in the separation of propylene/propane mixtures	2008
Gascon, J., Blom, W., Miltenburg, A., Ferreira, A., Berger, R., Kapteijn, F.	Netherlands	Accelerated synthesis of all-silica DD3R and its performance in the separation of propylene/propane mixtures	2008
Huang, J., Luo, H., Liang, C., Jiang, D., Dai, S.	China	Advanced Liquid Membranes Based on Novel Ionic Liquids for Selective Separation of Olefin/Paraffin via Olefin-Facilitated Transport	2008
Huang, J., Luo, H., Liang, C., Jiang, D., Dai, S.	China	Advanced Liquid Membranes Based on Novel Ionic Liquids for Selective Separation of Olefin/Paraffin via Olefin-Facilitated Transport	2008
Visser, T., Wessling, M.	Netherlands	Auto and mutual plasticization in single and mixed gas C3 transport through Matrimid-based hollow fiber membranes	2008
Kang, S. W., Honga, J., Char, K., Kimb, J. H., Kim, J., Kang, Y. S.	South Korea	Correlation between anions of ionic liquids and reduction of silver ions in facilitated olefin transport membranes	2008
Lee,J.S., Ko,N.H., Bae,H.W., Nguyen,D.Q., Lee,H., Choi, D.K., Cheonga,M., Kima,H.S.	South Korea	Effect of ester group on the performance of zwitterionic imidazolium compounds as membrane materials for separating alkene/alkane mixtures	2008
Kang,S.W., Lee,D.H., Park,J.H., Char,K., Kim,J.H., Won,J., Kang,Y.S.	South Korea	Effect of the polarity of silver nanoparticles induced by ionic liquids on facilitated transport for the separation of propylene/propane mixtures	2008
Ravanchi, M., Kaghazchi, T., Kargari, A.	Iran	Immobilized liquid membrane for propylene propane separation	2008
Kang, S. W., Hong, J., Park, J.H., Mun, S.H., Kim, J.H., Choe, J., Char, K., Kang, Y.S.	South Korea	Nanocomposite membranes containing positively polarized gold nanoparticles for facilitated olefin transport	2008
Kang, S.W., Char, K., Kang, Y.S.	South Korea	Novel Application of Partially Positively Charged Silver Nanoparticles for Facilitated Transport in Olefin/Paraffin Separation Membranes	2008
Ortiz, A., Ruiz, A., Gorri, D., Ortiz, I.	Spain	Room temperature ionic liquid with silver salt as efficient reaction media for propylene/propane separation: Absorption equilibrium	2008
Lee, D. H., Kang, Y. S., Kim, J. H., Kang, S. W.	South Korea	Selective Coordination of Silver Ions to Poly(styrene-b-(ethylene-co-butylene)-b-styrene) and its Influence on Morphology and Facilitated Olefin Transport	2008
Azhin, M., Kaghazchi, T., Rahmani, M.	Iran	A review on olefin/paraffin separation using reversible chemical complexation technology	2008
Hartmann, M., Himsl, D., Kunz, S., Tangermann, O.	Germany	Olefin/paraffin separation over the Metal Organic Framework material Cu3(BTC)2	2008
Ravanchi, M. T., Kaghazchi, T., Kargari, A., Soleimani, M.	Iran	A novel separation process for olefin gas purification: Effect of operating parameters on separation performance and process optimization	2009
Lamiaa, N., Jorge, M., Granato, M. A., Almeida Paz, F. A., Chevreau, H., Rodrigues, A. E.	Portugal	Adsorption of propane, propylene and isobutane on a metal–organic framework: Molecular simulation and experiment	2009
Ravanchi, M. T., Kaghazchi, T., Kargari, A.	Iran	Application of membrane separation processes in petrochemical industry: a review	2009
Hasan, R., Scholes, C.A., Stevens, G.W., Kentish, S.E.	Australia	Effect of Hydrocarbons on the Separation of Carbon Dioxide From Methane through a Polyimide Gas Separation Membrane	2009
Muna, S.H., Kang, S.W., Choc,J.S., Koh,S.K., Kanga,Y.S.	South Korea	Enhanced olefin carrier activity of clean surface silver nanoparticles for facilitated transport membranes	2009
Chng,M.L. Xiao,Y., Chunga,T.,Toriida,M.,Tamai, S.	Singapore	Enhanced propylene/propane separation by carbonaceous membrane derived from poly (aryl ether ketone)/2,6-	2009

		bis(4-azidobenzylidene)-4-methyl-cyclohexanone interpenetrating network	
Kim,J., Kang,S.W., Mun,S.H., Kang, Y.S.	South Korea	Facile Synthesis of Copper Nanoparticles by Ionic Liquids and Its Application to Facilitated Olefin Transport Membranes	2009
Das, M.	USA	Membranes for Olefin/Paraffin Separations	2009
Sánchez, L.M.G., Meindersma, G.W., Haan, A.B.	Netherlands	Potential of Silver-Based Room-Temperature Ionic Liquids for Ethylene/Ethane Separation	2009
Ravanchi, M.T., Kaghazchi, T., Kargari, A.	Iran	Separation of Propylene-Propane Mixture Using Immobilized Liquid Membrane via Facilitated Transport Mechanism	2009
Koha, J.H., Kang, S.W., Parka, J.T., Seoa, J.A., Kima,J.H., Kang,Y.S.	USA	Synthesis of silver halide nanocomposites templated by amphiphilic graft copolymer and their use as olefin carrier for facilitated transport membranes	2009
Li, K., Olson, D.H., Seidel, J., Emge,T.J., Gong,H., Zeng,H.,Li,J.	China	Zeolitic Imidazolate Frameworks for Kinetic Separation of Propane and Propene	2009
Gücüyener,C., Bergh,J.V.D.,Gascon,J., Kapteijn,F.	Netherlands	Ethane/Ethene Separation Turned on Its Head: Selective Ethane Adsorption on the Metal-Organic Framework ZIF-7 through a Gate-Opening Mechanism	2010
Rajabzadeha,S., Teramotoa,M., Al-Marzouqib, M. H., Kamioa, E., Ohmukaia, Y., Maruyamaa,T., Matsuyamaa,H.	Japan	Experimental and theoretical study on propylene absorption by using PVDF hollow fiber membrane contactors with various membrane structures	2010
Ravanchi,M.T., Kaghazchi,T., Kargari,A.	Iran	Facilitated transport separation of propylene–propane: Experimental and modeling study.	2010
Junga,S., Palgunadi, J., Kima,J.H., Lee,H.,Ahn,B.S.,Cheonga,M., Kim, H.S.	South Korea	Highly efficient metal-free membranes for the separation of acetylene/olefin mixtures: Pyrrolidinium-based ionic liquids as acetylene transport carriers	2010
Agel,F., Pitsch, F., Krull, F.F., Schulz,P.,Wessling,M., Melinb,T., Wasserscheida,P.	Germany	Ionic liquid silver salt complexes for propene/propane separation	2010
Das, M., Koros, W.J.	USA	Performance of 6FDA–6FpDA polyimide for propylene/propane separations	2010
Sadrzadeh, M., Shahidi, K., Mohammadi, T.	Iran	Preparation and C3H8/Gas Separation Properties of a Synthesized Single Layer PDMS Membrane	2010
Ortiz, A., Galan, L.M., Gorri, D., Haan, A.B. de, Ortiz, I.	Spain	Reactive Ionic Liquid Media for the Separation of Propylene/Propane Gaseous Mixtures	2010
Shi, M., Lin, C. C. H., Kuznicki, T. M., Hashisho, Z., Kuznicki, S. M.	Canada	Separation of a binary mixture of ethylene and ethane by adsorption on Na-ETS-10	2010
Ravanchi, M.T., Kaghazchi, T., Kargari, A.	Iran	Supported liquid membrane separation of propylene–propane mixtures using a metal ion carrier	2010
Chen J., Eldridge, R.B.,Rosen, E.L., Bielawski, C.W.	USA	A Study of Cu(I)-Ethylene Complexation for Olefin–Paraffin Separation	2011
Zou, X., Zhang, F., Thomas, S., Zhu, G., Vltchev, V., Mintova, S.	France	Co3(HCOO)6 Microporous Metal–Organic Framework Membrane for Separation of CO2/CH4 Mixtures	2011
Buxa, H., Chmelikb, C., Krishnac, R., Caro, J.	Germany	Ethene/ethane separation by the MOF membrane ZIF-8: Molecular correlation of permeation, adsorption, diffusion	2011
Zhang, C., Dai, Y., Johnson, J.R.,Karvan,O., Koros, W.J.	USA	High performance ZIF-8/6FDA-DAM mixed matrix membrane for propylene/propane separations	2011
Xu, L., Rungta, M.,Koros, W.J.	USA	Matrimid® derived carbon molecular sieve hollow fiber membranes for ethylene/ethane separation	2011
Bux, H., Feldhoff, A., Cravillon, J., Wiebcke, M., Li, Y., Caro, J.	Germany	Oriented Zeolitic Imidazolate Framework-8 Membrane with Sharp H2/C3H8 Molecular Sieve Separation	2011
Kang, S.W., Kang, Y.S.	South Korea	Silver nanoparticles stabilized by crosslinked poly(vinyl pyrrolidone) and its application for facilitated olefin transport	2011
Ferreira, A.F.P., Santos, J.C., Plaza, M.G., Lamia,N.,	Portugal	Suitability of Cu-BTC extrudates for propane–propylene separation by adsorption processes	2011

Loureiro, J.M., Rodrigues, A.E.			
Khoshkama, M., Sadeghib, M., Chenara, M.P., Fard, M.J.N., Baghersad, S.	Iran	Synthesis of polyimide membrane for the separation of CO2/CH4 gases	2011
Wang, Y., Ren, J., Deng, M.	China	Ultrathin solid polymer electrolyte PEI/Pebax2533/AgBF4 composite membrane for propylene/propane separation	2011
Aguado, S., Bergeret, G., Daniel, C., Farrusseng, D.	France	Absolute Molecular Sieve Separation of Ethylene/Ethane Mixtures with Silver Zeolite A	2012
Pan,Y., Li,T., Lestari,G., Lai, Z.	Saudi Arabia	Effective separation of propylene/propane binary mixtures by ZIF-8 membranes	2012
Yang, D., Currier, R.P., Le, L.A., Welch, C.F., Tornga,S.C., Martinez, R., Morrison, M.	USA	Enhanced Separation Efficiency in Olefin/Paraffin Distillation	2012
Fallanza, M., Ortiz, A., Gorri, D., Ortiz, I.	Spain	Experimental study of the separation of propane/propylene mixtures by supported ionic liquid membranes containing Ag^+–RTILs as carrier	2012
Lee, J.H., Kang, S.W., Song, D., Wond, J., Kang, Y.S.	South Korea	Facilitated olefin transport through room temperature ionic liquids for separation of olefin/paraffin mixtures	2012
Kim, J.,Lin, L., Martin, R.L., Swisher, J.A., Haranczyk,M., Smit, B.	USA	Large-Scale Computational Screening of Zeolites for Ethane/Ethene Separation	2012
Shamsabadi, A.A., Kargari, A., Farshadpour, F., Laki, S.	Iran	Mathematical Modeling of CO2/CH4 Separation by Hollow Fiber Membrane Module Using Finite Difference Method	2012
Rungta, M., Xu, L., Koros, W. J.	USA	Carbon molecular sieve dense film membranes derived from Matrimid® for ethylene/ethane separation	2012
Motelica, A., Bruinsma, O.S.L., Kreiter, R., Den Exter, M., Vente, J.F.	Netherlands	Membrane Retrofit Option for Paraffin/Olefin Separation - A Technoeconomic Evaluation	2012
Askari, M., Xiao, Y., Li, P., Chung, T.	Singapore	Natural gas purification and olefin/paraffin separation using cross-linkable 6FDA-Durene/DABA co-polyimides grafted with a, b , and g -cyclodextrin	2012
Askari, M., Yang, T.X., Chung, T.	Singapore	Natural gas purification and olefin/paraffin separation using cross-linkable dual-layer hollow fiber membranes comprising b-Cyclodextrin	2012
Xu,L., Rungt, M., Brayden, M.K., Martinez, M.V., BrienA.Stears,B.A., Barbay,G.A., Koros, W.J.	USA	Olefins-selective asymmetric carbon molecular sieve hollow fiber membranes for hybrid membrane-distillation processes for olefin/paraffin separations	2012
Faiz, R., Fallanza, M., Ortiz, I., Li, K.	UK	Olefin/Paraffin Separation using Ceramic Hollow Fiber Membrane Contactors	2012
Faiz, R., Li, K.	UK	Olefin/paraffin separation using membrane based facilitated transport/chemical absorption techniques	2012
Kanezashi,M., Kawano,M., Yoshioka,T., Tsuru,T.	Japan	Organic Inorganic Hybrid Silica Membranes with Controlled Silica Network Size for Propylene/Propane Separation	2012
Pollo, L.D., Duarte,L.T., Anacleto, M., Habert,A.C., Borges, C.P.	Brasil	Polymeric Membranes Containing Silver Salts for Propylene/Propane Separation	2012
Faiz, R., Li, K.	UK	Polymeric membranes for light olefin/paraffin separation	2012
Kuraoka, K., Matsuura, S., Ueda, K.	Japan	Preparation and properties of organic - inorganic hybrid facilitated olefin separation membranas via sol-gel method	2012
Naghsh,M., Sadeghi, M., Moheb,A., Chenar,M.P., Mohagheghian,M.	Iran	Separation of ethylene/ethane and propylene/propane by cellulose acetate–silica nano composite membranes	2012
Kanezashi, M., Shazwani,W.N., Yoshioka, Tsuru,T.	Japan	Separation of propylene/propane binary mixtures by bis(triethoxysilyl) methane (BTESM)-derived silica membranes fabricated at different calcination temperatures.	2012
Saedi, S., Madaeni, S.S., Shamsabadi, A.A., Mottaghi, F.	Iran	The effect of surfactants on the structure and performance of PES membrane for separation of carbon dioxide from methane	2012
Bloch,E.D., Queen,W.L.,Krishna,R.,	USA	Hydrocarbon Separations in a Metal-Organic Framework with Open Iron(II) Coordination Sites	2012

Zadrozny,J.M.,Brown,C.M., Long,J.R.			
Kasahara S., Kamio, E., Minami, R., Matsuyama, H.	Japan	A facilitated transport ion-gel membrane for propylene/propane separation using silver ion as a carrier	2013
Ploegmakers, J., Jelsma, A.R.T., Van der Ham, A.G., Nijmeijer, K.	Netherlands	Economic Evaluation of Membrane Potential for Ethylene/Ethane Separation in a Retrofitted Hybrid Membrane-Distillation Plant Using Unisim Design	2013
Böhme, U., Barth, B., Paula, C., Mundstock, A., Caro, J., Hartmann, M.	Germany	Ethene/Ethane and Propene/Propane Separation via the Olefin and Paraffin Selective Metal−Organic Framework Adsorbents CPO-27 and ZIF-8	2013
Kuraoka, K., Matsuura, S., Ueda, K.	Japan	Ethylene/Ethane Separation through a SiO2 Poly(sodium acrylate) Ag^+ Organic Inorganic Hybrid Membrane	2013
Hong, G.H., Ji, D., Kang, S.W.	South Korea	Highly Permeable Ionic Liquid/Cu Composite Membrane for Olefin/Paraffin Separation	2013
Yang,D.l., Le,L. ,Martinez, R.	USA	Hollow Fibers Structured Packings in Olefin/Paraffin Distillation: Apparatus Scale-Up and Long-Term Stability	2013
Kwon,H.T.,Jeong, H.	USA	In Situ Synthesis of Thin Zeolitic−Imidazolate Framework ZIF-8 Membranes Exhibiting Exceptionally High Propylene/Propane Separation	2013
Faiz, R., Fallanza, M.,Boributh, S., Jiraratananon, R., Ortiz, I., Li , K.	UK	Long term stability of PTFE and PVDF membrane contactors in the application of propylene/propane separation using AgNO3 solution	2013
Rungta, M., Zhang, C., Xu, L., Koros, W.	USA	Membrane-based Ethylene/Ethane Separation: The Upper Bound and Beyond	2013
Ploegmakers, J., Japip, S., Nijmeijer, K.	Netherlands	Mixed matrix membranes containing MOFs for ethylene/ethane separation Part A: Membrane preparation and characterization	2013
Askari, M., Chung, T.	Singapore	Natural gas purification and olefin/paraffin separation using thermal cross-linkable co-polyimide/ZIF-8 mixed matrix membranes	2013
Brayden, M., Koros, W., Xu, L., Martinez, M., Stears, B., Barbay, G.	USA	Carbon Molecular Sieve Hollow Fiber Membranes for Olefin/Paraffin Separations	2013
Ma, X., Lin, B., Wei, X., Kniep, J., Lin, Y.	USA	Gamma-Alumina Supported Carbon Molecular Sieve Membrane for Propylene/Propane Separation	2013
Fallanza, M., Ortiz, A., Gorri, D., Orti.I.z	Spain	Polymer−ionic liquid composite membranes for propane/propylene separation by facilitated transport	2013
Huang, L., Cao, D.	China	Selective adsorption of olefin–paraffin on diamond-like frameworks: diamondyne and PAF-302	2013
Faiz, R., Fallanza, M., Ortiz, I., Li, K.	UK	Separation of Olefin/Paraffin Gas Mixtures Using Ceramic Hollow Fiber Membrane Contactors	2013
Merkel, T.C., Blanc, R., Ciobanu, I., Firat, B., Suwarlim, A.,Zeid, J.	USA	Silver salt facilitated transport membranes for olefin/paraffin separations: Carrier instability and a novel regeneration method	2013
Hamza, A.A., Towe, I.G., Keyvani, M.	USA	Stable Facilitated Transport Membrane for Olefin/Paraffin Separation	2013
Saedi, S., Madaeni, S.S., Hassanzadeh, K., Shamsabadi, A.A., Laki, S.	Iran	The effect of polyurethane on the structure and performance of PES membrane for separation of carbon dioxide from methane	2013
Fallanza, M., Ortiz, A., Gorri, D., Ortiz, I.	Spain	Using Membrane Reactive Absorption Modeling to Predict Optimum Process Conditions in the Separation of Propane−Propylene Mixtures	2013
Ploegmakers, J., Japip, S., Nijmeijer, K.	Netherlands	Mixed matrix membranes containing MOFs for ethylene/ethane separation—Part B: Effect of Cu3BTC2 on membrane transport properties	2013
Ni, H., Hsu, C.S., Ma, C., Shi, Q., Xu, C.	China	Separation and Characterization of Olefin/Paraffin in Coal Tar and Petroleum Coker Oil	2013
Li, P., He, Y., Arman, H. D., Krishna, R., Wang, H., Wengc, L., Chen, B.	USA	A microporous six-fold interpenetrated hydrogenbonded organic framework for highly selective separation of C2H4/C2H6	2014
Bandehali, S., Kargari, A., Moghadassi, A., Saneepur, H., Ghanbari, D.	Iran	Acrylonitrile–butadiene–styrene/poly(vinyl acetate)/nanosilica mixed matrix membrane for He/CH4 separation	2014
Ismail, N.M., Islmail, A.F., Mustaffa, A.	Malaysia	Characterization of Polyethersulfone/Cloisite 15A Mixed Matrix Membrane for CO2/CH4 Separation	2014
Fallanza, M., Ortiz, A., Gorri, D., Ortiz, I.	Spain	Effect of liquid flow on the separation of propylene/propane mixtures with a gas/liquid membrane contactor using Ag^+-RTIL solutions	2014

Pires,J., Pinto,M.L., Saini, V.K.	Portugal	Ethane Selective IRMOF-8 and Its Significance in Ethane−Ethylene Separation by Adsorption	2014
Chang, G., Bao, Z., Ren, Q., Su, B., Xinga, H., Yanga, Y.	USA	Fabrication of cuprous nanoparticles in MIL-101: an efficient adsorbent for the separation of olefin–paraffin mixtures	2014
Ghasem, N., Al-Marzouqi, M., Ismail, Z.	United Arab Emirates	Gas–liquid membrane contactor for ethylene/ethane separation by aqueous silver nitrate solution	2014
Grande,C.A., Lind,A., Vistad,Ø., Akporiaye, D.	Norway	Olefin−Paraffin Separation Using Calcium-ETS-4	2014
Shahid,S., Nijmeijer,K.	Netherlands	Performance and plasticization behavior of polymer–MOF membranes for gas separation at elevated pressures	2014
Tome, L.C., Mecerreyes, D., Freire, C.S.R., Rebeloa, L.P.N., Marrucho, I.M.	Portugal	Polymeric ionic liquid membranes containing IL–Ag^+ for ethylene/ethane separation via olefin-facilitated transport	2014
Lei, G., Liu, C., Xie, H., Song, F.	China	Separation of the hydrogen sulfide and methane mixture by the porous graphene membrane: Effect of the charges	2014
Sun, H., Ma, C., Wang, T., Xu, Y., Yuan, B., Kong, Y.	China	Satellite TiO2 nanoparticles induced by silver ion in polymer electrolytes membrane for propylene/propane separation	2014
Kemenade, H.P., Benthun, R.J., Brouwers, J.J.H.	Netherlands	Upgrading Carbon Dioxide/Methane Mixtures by using a Hybrid Membrane–Condensed Rotational Separation Process	2014
Wang,Y., Thompson,J., Zhou,J., Goodrich,P., Atilhan,M., Pensado,A.S., Kirchner,B., Rooney,D., Jacquemin,J., Khraisheh,M.	Qatar	Use of water in aiding olefin/paraffin (liquid + liquid) extraction via complexation with a silver bis(trifluoromethylsulfonyl)imide salt	2014
Li, L.S., Yeong, Y.F., Lau, K.K., Azmi, M.S.	Malaysia	Zeolitic Imidazolate Frameworks (ZIF): A Potential Membrane for CO2/CH4 Separation	2014
Yeo, Z.Z., Chai, S., Zhu, P.W., Mohamed, A.R.	Malaysia	Development of a hybrid membrane through coupling of high selectivity Zeolite T on ZIF-8 intermediate layer and its performance in carbon dioxide and methane gas separation	2014
Pedram, S., Kaghazchi, T., Ravanchi, M.T.	Iran	Performance and Energy Consumption of Membrane-Distillation Hybrid Systems for Olefin-Paraffin Separation	2014
Chen, D., Shang, H., Zhu, W., Krishna, R.	Netherlands	Transient breakthroughs of CO2/CH4 and C3H6/C3H8 mixtures in fixed beds packed with Ni-MOF-74	2014
Hartmann, M., Bo̎hme, U., Hovestadt, M., Paula, C.	Germany	Adsorptive Separation of Olefin/Paraffin Mixtures with ZIF-4	2015
Najaria, S., Omidkhaha, M., Hosseinia, S.S.	Iran	An Investigation on the Factors Affecting the Properties and Performance of Polymeric Nanocomposite Membranes for Olefin/Paraffin Separation	2015
Adewole, J.K., Ahmad, A.L., Ismail, S., Leo, C.P., Sultan, A.S.	Malaysia	Comparative studies on the effects of casting solvent on physicochemical and gas transport properties of dense polysulfone membrane used for CO2/CH4 separation	2015
Hamza, A.A., Martin, J., Barbouti, M., Rosales, C., Perez, J.C., Towe, I.G.	USA	Continuous Olefin/Paraffin Separation with PermyleneTM Facilitated Transport Membranes from Imtex Membranes Corp.	2015
Ravanchi, M.T., Kaghazchi, T., Kargari, A.	Iran	Effect of Complexation Reaction Constant on the Separation of Propylene/Propane by Supported Liquid Membrane	2015
Swaidan, R., Ma., X., Litwiller, E., Pinnau, I.	Saudi Arabia	Enhanced propylene/propane separation by thermal annealing of an intrinsically microporous Hydroxylfunctionalized polyimide membrane	2015
Wang,Y., Hao, W., Jacquemin,J., Goodrich,P., Atilhan,M., Khraisheh M., Rooney,D.,Thompso, J.	Qatar	Enhancing Liquid-Phase Olefin−Paraffin Separations Using Novel Silver-Based Ionic Liquids	2015
Deng, L., Hägg, M.	Norway	Fabrication and evaluation of a blend facilitated transport membrane for CO2/CH4 separation	2015
Wiheeba, A.D., Kim, J., Othman, M.R.	Malaysia	Highly perm-selective micro-porous hydrotalcite-silica membrane for improved carbon dioxide-methane separation	2015
Chew, T.L., Ahmad, A.L., Bhatia, S.	Malaysia	Microwave heating-synthesized zeolite membrane for CO2/CH4 separation	2015

Liu, J., Chen, X., Zhao, S., Cao, X., Shen, B.	China	Multicycle Investigation of Normal Paraffin Separation from Naphtha To Improve Olefin and Aromatic Feed	2015
McCullough, D.W., Kapur, S.	USA	Petrochemical Technology Trends: Looking Beyond the Short Term Fix	2015
Najari,S., Hosseini, S.S., Omidkhaha, M.,Tan, N.R.	Iran	Phenomenological modeling and analysis of gas transport in polyimide membranes for propylene/ propane separation	2015
Sun, H., Yuan, B., Li, P.	China	Preparation of nanoporous graphene and the application of its nanocomposite membrane in propylene/propane separation	2015
Zheng, Y.,Hu, N., Wang, H., Bu,N., Zhang,F., Zhou,R.	China	Preparation of steam-stable high-silica CHA (SSZ-13) membranes for CO_2/CH_4 and C_2H_4/C_2H_6 separation	2015
Azizi, S., Kaghazchi, T., Kargari, A.	Iran	Propylene/propane separation using N-methyl pyrrolidone/$AgNO_3$ supported liquid membrane	2015
Salinas,O., Ma,X., Litwiller,E., Pinnau, I.	Saudi Arabia	High-performance carbon molecular sieve membranes for ethylene/ethane separation derived from an intrinsically microporous polyimide	2015
Murali,R.S., Rani,K.Y., Sankarshana,T., Ismail,A.F., Sridhar,S.	India	Separation of Binary Mixtures of Propylene and Propane by Facilitated Transport through Silver Incorporated Poly(Ether-Block-Amide) Membranes	2015
Ma, X., Williams, S., Wei, S., Kniep, J., Lin,Y.S.	USA	Propylene/Propane Mixture Separation Characteristics and Stability of Carbon Molecular Sieve Membranes	2015
Sun, Q., Qi, B., Liu, A., Guo, X., Zhang, J.	China	Separation of H_2/CH_4 Through TBAB Hydrate Membrane	2015
Islamil, N.H., Salleh, W.N.W., Sazali, N., Ismail, A.F.	Malaysia	The Effect of Polymer Composition on CO_2/CH_4 Separation of Supported Carbon Membrane	2015
Cadiau, A., Adil, K., Bhatt, P. M., Belmabkhout, Y., Eddaoudi, M.	USA	A metal-organic framework–based splitter for separating propylene from propane	2016
Mei, L., Wu, Y., Zhou, X., Yan, J., Xu, F., Li, Z.	China	Adsorption performance of MIL-100(Fe) for separation of olefin–paraffin mixtures	2016
Jusoh, N., Lau, K.K., Yeong, Y.F., Shariff, A.M.	Malaysia	Bulk CO_2/CH_4 Separation for Offshore Operating Conditions using Membrane Process	2016
Maghsoud, H.	Iran	Comparative study of adsorbents performance in ethylene/ethane separation	2016
Mohshim, D.F., Mukhtar, H., Man, Z.	Malaysia	Composite blending of ionic liquid–poly(ether sulfone) polymeric membranes: Green materials with potential for carbon dioxide/methane separation	2016
Suleman, M.S., Lau, K.K., Yeong, Y.F.	Malaysia	Development and performance evaluation of Polydimethyl siloxane/Polysulfone (PDMS/PSF) composite membrane for CO_2/CH_4 separation	2016
Park, Y.S., Chuna, S., Kang, Y.S., Kang, S.W.	South Korea	Durable poly(vinyl alcohol)/$AgBF_4/Al(NO_3)_3$ complex membrane with high permeance for propylene/propane separation	2016
Mohamad, M.B., Fong, Y.Y., Shafirff, A.	Malaysia	Gas Separation of Carbon Dioxide from Methane using Polysulfone Membrane Incorporated with Zeolite-T	2016
Doğu,M.,Ercan,N.	Turkey	High performance cyclic olefin copolymer (COC) membranes prepared with melt processing method and using of surface modified graphitic nanosheets for H_2/CH_4 and H_2/CO_2 separation	2016
Liao, K., Lai, J., Chung, T.	Taiwan	Metal ion modified PIM-1 and its application for propylene/propane separation	2016
Park, C.H., Lee, H.H., Jung, J.P., Kim, J.H.	South Korea	Mixed Matrix Membranes Based on Dualfunctional MgO Nanosheets for Olefin/Paraffin Separation	2016
Hamrahi, Z., Kargari, A.	Iran	Modification of polycarbonate membrane by polyethylene glycol for CO_2/CH_4 separation	2016
Shokrian, M., Sadrzadeh, M., Mohammadi, T.	Iran	Neural Network Modelling of C_3H_8 separation from CH_4 & H_2 using PDMS membrane	2016
Bendt, S., Hovestadt, M., Döpken, M., Hartmann, M., Keil, F.J.	Germany	Olefin/Paraffin Separation Potential of ZIF-9 and ZIF-71: A Combined Experimental and Theoretical Study	2016
Isanejad, M., Azizi, N., Mohammadi, T.	Iran	Pebax Membrane for CO_2/CH_4 Separation: Effects of Various Solvents on Morphology and Performance	2016
Saedi, S., Saedi, F., Moradi, F., Xiang, X.	USA	Preparation and characterization of an amino-cellulose (AC) derivative for development of thin-film composite membrane for CO_2/CH_4 separation	2016
Kulkarni, A. R., Sholl, D. S.	USA	Screening of Copper Open Metal Site MOFs for Olefin/Paraffin Separations Using DFT-Derived Force Fields	2016
Kim, S., Ko, D., Row, S., Kim, J.	South Korea	Techno-economic evaluation of hybrid systems of pressure swing adsorption and membrane processes for	2016

		coalbed methane separation	
Ma, X., Lin, Y., Wei, X., Kniep, J.	USA	Ultrathin Carbon Molecular Sieve Membrane for Propylene/Propane Separation	2016
Zahri, K., Goh, P.S., Ismail, A.F.	Malaysia	The incorporation of graphene oxide into polysulfone mixed matrix membrane for CO2/CH4 separation	2016
Eum,K., Ma,C., Rownaghi,A., Jones,C.W., Nair,S.	USA	ZIF-8 Membranes via Interfacial Microfluidic Processing in Polymeric Hollow Fibers: Efficient Propylene Separation at Elevated Pressures	2016
Yongli Sun,†,‡ Hanrong Bi,†,‡ Haozhen Dou,†,‡ Huawei Yang,†,‡ Zhaohe Huang,†,‡ Baoyu Wang,†,‡ Rong Deng,†,‡ and Luhong Zhang*,†,‡	China	A Novel Copper(I)-Based Supported Ionic Liquid Membrane with High Permeability for Ethylene/Ethane Separation	2017
Bin Jiang, Wenjun Tao, Haozhen Dou, Yongli Sun, Xiaoming Xiao, Luhong Zhang, and Na Yang	China	A Novel Supported Liquid Membrane Based on Binary Metal Chloride Deep Eutectic Solvents for Ethylene/Ethane Separation	2017
Rong Deng,† Yongli Sun,*,† Hanrong Bi,† Haozhen Dou,† Huawei Yang,† Baoyu Wang,† Wenjun Tao,† and Bin Jiang†	China	Deep Eutectic Solvents As Tuning Media Dissolving Cu^+ Used in Facilitated Transport Supported Liquid Membrane for Ethylene/ Ethane Separation	2017
Gamali, P.A., Kazemi, A., Zadmard, R., Anjareghi, M.J., Rezakhani, A., Rahighi, R., Madani, M.	Iran	Distinguished discriminatory separation of CO2 from its methane-containing gas mixture via PEBAX mixed matrix membrane	2017
Young Sung Park a, Soonwoo Chun a, Yong Soo Kang b,⇑, Sang Wook Kang a,⇑	South Korea	Durable poly(vinyl alcohol)/AgBF4/Al(NO3)3 complex membrane with high permeance for propylene/propane separation	2017
Jeong, S., Kang, S.W.	South Korea	Effect of Ag2O nanoparticles on long-term stable polymer/AgBF4/Al(NO3)3 complex membranes for olefin/paraffin separation	2017
Luna-Triguero, A., Vincent-Luna, J.M., Becker, T.M., Vlugt, T.J.H., Dubbeldam, D., Alvarez, P.G., Calero, S.	Spain	Effective Model for Olefin/Paraffin Separation using (Co,Fe, Mn, Ni)-MOF-74	2017
Brayden, M., Xu, L., Barbay, G., Koros, W.J.	USA	Impact of Impurities on Carbon Molecular Sieve Membranes for Application in Olefins Units	2017
Chu, Y., Koros, W.J., Yancey, D.F., Brayden, M., Martinez, M., Xu, L.	USA	Iron-containing Carbon Molecular Sieve Membranes for Advanced Olefin/Paraffin Separations	2017
Han, Y.J., Ko, K.J., Choi, H.K., Moon, J.H., Lee, C.H.	South Korea	Kinetic effects of methane on binary mixture separation on methyltriethoxysilane templated silica membranes	2017
Khoshkaram, A., Ghayyem, M.A., Behbahani, R.M.	Iran	Laboratory investigation of carbon dioxide separation from methane using a PES/Pebax 1657 composite membrane	2017
Kovács, T., Papp, S., Kristóf, T.	Hungary	Membrane separation study for methane-hydrogen gas mixtures by molecular simulations	2017
Jiang, B., Dou, H., Zhang, L., Wang, B., Sun, Y., Yang, H., Huang,Z., Bi, H.	China	Novel supported liquid membranes based on deep eutectic solvents for olefin-paraffin separation via facilitated transport	2017
Luna-Triguero, A., Vincent-Luna, Gómez-Álvarez, P., Calero, S.	Spain	Olefin/Paraffin Separation in Open Metal Site Cu-BTC Metal-Organic Framework	2017
Majumdar, S., Koizumi, Y., Loprete, K., Pennisi, K., Feiring, A., Shangguan, N., Murnen, H., Lousenberg, D.	USA	Olefin-Paraffin Separation with Customized Amorphous Fluoropolymer (CAF) Facilitated Transport Membranes	2017
Park, J., Kim, K., Shin, J., Tak, K., Park, Y.	South Korea	Performance Study of Multistage Membrane and Hybrid Distillation Processes for Propylene/Propane Separation	2017
Ovcharova, A., Vasilevsky, V., Borisov, I.,	Russia	Polysulfone porous hollow fiber membranes for ethylene-ethane separation in	2017

Bazhenov, S., Volkov, A., Bildyukevich, A., Volkov, V.		gas-liquid membrane contactor	
Jiang, C., Hou, Y., Wang, N., Li, L., Lin, L., Niu, Q.J.	China	Propylene/propane separation by porous graphene membrane: Molecular dynamic simulation and first-principle calculation	2017
Han, Y.J., Kang, J.H., Kim, H.E., Moon, J.H., Cho, C.H., Lee, C.H.	South Korea	Separation of Carbon Dioxide and Methane Mixture by an Adsorbent/Membrane Hybrid (AMH) System using Zeolite 5A Pellets and FAU-Zeolite Membrane	2017
Jiang,B., Dou, H., Wang,B., Sun,Y., Huang,Z., Bi, H., Zhang,L., Yang, H.	China	Silver-based deep eutectic solvents as separation media: supported liquid membranes for facilitated olefin transport	2017
Lai, L.S., Yeong, Y.F., Lau, K.K., Shariff, A.M.	Malaysia	Single and Binary CO2/CH4 Separation of Zeolitic Imidazolate Framework-8 Membrane: Experimental and Modeling Study	2017
Chultheera, P., Rirksomboon, T., Kulprathipanja, S., Liu, C., Chinsirikul, W., Kerddonfag, N.	Thailand	Solid-Liquid-Polymer Mixed Matrix Membrane Using Liquid Additive Adsorbed on Activated Carbon Dispersed in Polymeric Membrane for CO2/CH4 Separation	2017
Lee, U., Kim, J., Chae, S., Han, C.	South Korea	Techno-economic feasibility study of membrane based propane/propylene separation process	2017
Yu, L., Grahn, M., Ye, P., Hedlund J.	Sweden	Ultra-thin MFI membranes for olefin/nitrogen separation	2017
Maximilian Hovestadt 1 ID , Sebastian Friebe 2, Lailah Helmich 2, Marcus Lange 3, Jens Möllmer 3, Roger Gläser 3 ID , Alexander Mundstock 2 and Martin Hartmann	Germany	Continuous Separation of Light Olefin/Paraffin Mixtures on ZIF-4 by Pressure Swing Adsorption and Membrane Permeation	2018
Jian Yu 1, Chongqing Wang1, Long Xiang, Yuanze Xu, Yichang Pan ⇑	China	Enhanced C3H6/C3H8 separation performance in poly(vinyl acetate) membrane blended with ZIF-8 nanocrystals	2018
Jung Pyo Jung,1 Min June Kim,2 Youn-Sang Bae,1,2 Jong Hak Kim	South Korea	Facile preparation of Cu(I) impregnated MIL-101(Cr) and its use in a mixed matrix membrane for olefin/paraffin separation	2018
Liang Ma, Frantisek Svec, Tianwei Tan, and Yongqin Lv	China	Mixed Matrix Membrane Based on Crosslinked Poly[(Ethylene Glycol) Methacrylate] and Metal-Organic Framework for Efficient Separation of Carbon Dioxide and Methane	2018
Hamid Reza Amedi , Masoud Aghajani	Iran	Modified zeolitic–midazolate framework 8/poly(ether-block-amide) mixed-matrix membrane for propylene and propane separation	2018
Sheng Zhou1*, Yanying Wei1*, Libo Li1*, Yifan Duan1, Qianqian Hou1, Lili Zhang1, Liang-Xin Ding1, Jian Xue1, Haihui Wang1†, Jürgen Caro2	China	Paralyzed membrane: Current-driven synthesis of a metal-organic framework with sharpened propene/ propane separation	2018
Hafez Maghsoudi	Iran	Theoretical screening of zeolites for membrane separation of propylene/propane mixtures	2018
Park J., Kim K., Shin J.-W., Park Y.-K.	South Korea	Analysis of Multistage Membrane and Distillation Hybrid Processes for Propylene/propane Separation	2019
Yang Liu, Zhijie Chen, Gongping Liu, Youssef Belmabkhout, Karim Adil, Mohamed Eddaoudi,* and William Koros*	USA	Conformation-Controlled Molecular Sieving Effects for Membrane-Based Propylene/Propane Separation	2019
Shaohua Chaia,b, Hongbin Dub, Yayun Zhaoa,b, Yichao Linb, Chunlong Kongb,⁎, Liang Chenb]	China	Fabrication of highly selective organosilica membrane for gas separation by mixing bis(triethoxysilyl)ethane with methyltriethoxysilane	2019
Sweta Shrestha, Prabir K. Dutta	USA	Modification of a continuous zeolite membrane grown within porous polyethersulfone with Ag(I) cations for enhanced propylene/propane gas separation	2019

Yingfei Hou, Yiyu Li, Chi Jiang, Yang Xu, Mumin Wang & Qingshan Jason Niu	China	Molecular simulation for separation of ethylene and ethane by functionalised graphene membrane	2019
Motomu Sakai, Yasuhito Sasaki, Taisuke Tomono, Masahiro Seshimo, and Masahiko Matsukata	Japan	Olefin Selective Ag-exchanged X-type Zeolite Membrane for Propylene/Propane and Ethylene/Ethane Separation	2019
Junjun Hou, Pengchao Liu, Meihuizi Jiang, Lian Yu, Lianshan Li* and Zhiyong Tang	China	Olefin/paraffin separation through membrane: from mechanisms to critical materials	2019
Raúl Zarca1, Alfredo Ortiz1, Daniel Gorri1, Lorenz T. Biegler2, Inmaculada Ortiz1	Spain	Optimization of Multistage Olefin/Paraffin Membrane Separation Processes through Rigorous Modeling	2019
Margarita Kostyanaya 1, Stepan Bazhenov 1, Ilya Borisov 1 , Tatiana Plisko 2 and Vladimir Vasilevsky	Russia	Surface Modified Polysulfone Hollow Fiber Membranes for Ethane/Ethylene Separation Using Gas-Liquid Membrane Contactors with Ionic Liquid-Based Absorbent	2019
A.O. Malakhova, S.D. Bazhenova,⁎, V.P. Vasilevskya, I.L. Borisova, A.A. Ovcharovaa, A.V. Bildyukevichb, V.V. Volkova, L. Giornoc, A.V. Volkova	Russia	Thin-film composite hollow fiber membranes for ethylene/ethane separation in gas-liquid membrane contactor	2019
